# The Effects of Surface Spin Polarization on Copper Oxidation by Triplet Oxygen

**DOI:** 10.1021/acsnano.5c21063

**Published:** 2026-02-11

**Authors:** Avi Schneider, Meital Ozeri, Yael Kapon, Ralfy Kenaz, Vitaly Gutkin, Shira Yochelis, Lech Tomasz Baczewski, Doron Azulay, Oded Millo, Yossi Paltiel

**Affiliations:** † Applied Physics Department and Center for Nano-Science and Nano-Technology, 26742 The Hebrew University of Jerusalem, Jerusalem 9190401, Israel; ‡ Racah Institute of Physics and the Hebrew University Center for Nanoscience and Nanotechnology, The Hebrew University of Jerusalem, Jerusalem 91904, Israel; § The Center for Nanoscience and Nanotechnology, The Hebrew University of Jerusalem, Jerusalem 91904, Israel; ∥ Institute of Physics Polish Academy of Sciences, Al. Lotnikow 32/46, Warszawa 02-668, Poland; ⊥ Department of Physics, Azrieli College of Engineering, Jerusalem 9103501, Israel

**Keywords:** metal oxidation, spin-dependent oxidation, surface spin effects, triplet oxygen reactivity, atomic force microscopy, Kelvin probe force microscopy

## Abstract

The process of copper oxidation has been thoroughly studied for many years, yielding a significant understanding of its kinetics and chemistry. However, the possible roles of surface spin polarization in important issues such as oxidation rates have not been widely explored despite the triplet nature of molecular oxygen. Here, we investigate the spin-dependent oxidation of copper films by triplet O_2_, exploiting engineered ferromagnetic substrates to impose controlled surface spin polarization. Three sample architectures that enable comparison between spin-polarized and nonpolarized surfaces were implemented to enable direct comparison between regions of varying spin polarization on the same sample. Combining various surface-sensitive techniques, including atomic force microscopy, Kelvin probe force microscopy, ellipsometry, and magneto-optical Kerr effect, we followed oxide growth kinetics and electronic property changes over time scales from minutes to weeks. Our results demonstrate that spin-polarized surfaces exhibit a significant acceleration in copper oxide formation compared with less polarized regions. The difference appears to be driven by a preference toward the formation of cupric oxide (CuO), the second oxidation state of copper, over cuprous oxide (Cu_2_O), the first oxidation state. We suggest that the results are related to the different magnetic properties of each oxide. Our data also reveal that the CuO oxidation phase propagates from the Cu film edges toward the center of the sample. These findings provide direct evidence of the surface-spin influence on metal oxidation kinetics and support the notion that spin polarization can induce a lower activation energy barrier for electron transfer between metal to triplet O_2_. Beyond advancing the fundamental understanding of corrosion chemistry, this spin-dependent control of surface reactivity opens potential avenues for tailored catalyst design, spintronic device stability, and corrosion mitigation strategies.

## Graphical abstract

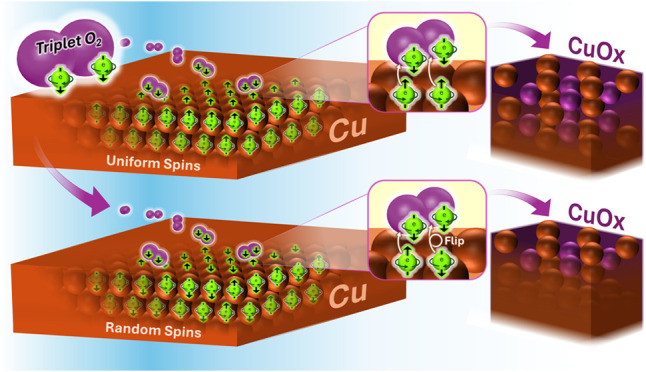

## 1 Introduction

From its first basic use in prehistoric tool making to its various modern-day uses, copper (Cu) is and has been a versatile, highly essential, and prevalent material in daily human life throughout the ages.^1^ However, although it is sometimes classified as a precious metal, elemental copper is highly prone to oxidation under any standard conditions.^2^ Copper oxidation is a ubiquitous and critical chemical process with wide-ranging technological applications. On one hand, uncontrolled copper oxidation poses a serious challenge in microelectronics, nanofabrication, and energy applications, due to its effects on electrical and thermal conductivity.^3,4^ On the other hand, the formation of a passivating copper oxide layer can help in preventing further corrosion and protect the integrity of the bulk metal, a property widely exploited in architectural materials and catalysis.^5^ Consequently, the process of copper oxidation has already been thoughtfully investigated, leading to an in-depth understanding of its kinetics and chemistry.^2^

At the molecular level, copper oxidation involves the transfer of electrons from the metal, acting as a reducing species, to an oxidizing agent, typically molecular oxygen (O_2_). The complete oxidation of copper to its Cu­(I) and then Cu­(II) states requires the donation of two electrons to the O_2_ molecules and results in the formation of cuprous oxide (Cu_2_O) and cupric oxide (CuO), respectively. The oxidation process involves an initial adsorption of O_2_ onto the surface, followed by electron transfer from the metal to the oxygen species. The progress of the process is then dependent on further diffusion of O_2_ through the formed oxide to unexposed metallic copper. This oxidation process is sensitive to environmental factors, to the crystallographic orientation of the Cu surface, as well as to the electronic properties of the surface atoms themselves.^2,6–10^

Due to the unique triplet nature of molecular oxygen, most electron transfer events in which it is involved must overcome a fundamental quantum constraint. Triplet oxygen has a ground-state spin configuration with two unpaired electrons (a triplet, *S* = 1), while most materials and reaction intermediates are singlets (*S* = 0). This feature introduces spin selection rules into the chemical reactivity of O_2_ and oxidation reactions. It may thus be spin-forbidden or slowed down and often require spin conversion processes or spin-aligned electron pairing in order to proceed efficiently.^11–13^

This raises the possibility that the spin state of electrons at the metal surface could influence the oxidation kinetics or mechanism. If the surface electrons are spin-polarized, then the alignment (or misalignment) of spins between the metal surface and the oxygen molecule could potentially alter the likelihood or pathway of oxidation.^14,15^ This concept has been explored in surface catalysis, magnetic thin films, and molecular spintronics, but remains largely untested in controlled metal oxidation systems.^16–19^ Addressing this gap is important because, as indicated above, electron spin alignment at the surface could directly influence oxidation pathways and rates, potentially affecting both fundamental understanding and practical applications of Cu and other metals.

Given this, it is reasonable to hypothesize that spin polarization at the Cu surface could influence the kinetics and mechanism of oxidation. As illustrated in Figure 1, if electrons on the copper surface are uniformly spin-aligned, this could change their availability to react with the spin-polarized oxygen molecules. This notion draws on concepts from spin-selective chemistry and chiral-induced spin selectivity (CISS), where spin filtering effects have been observed to impact charge transfer and chemical reactions.^18–21^

**Figure 1:**
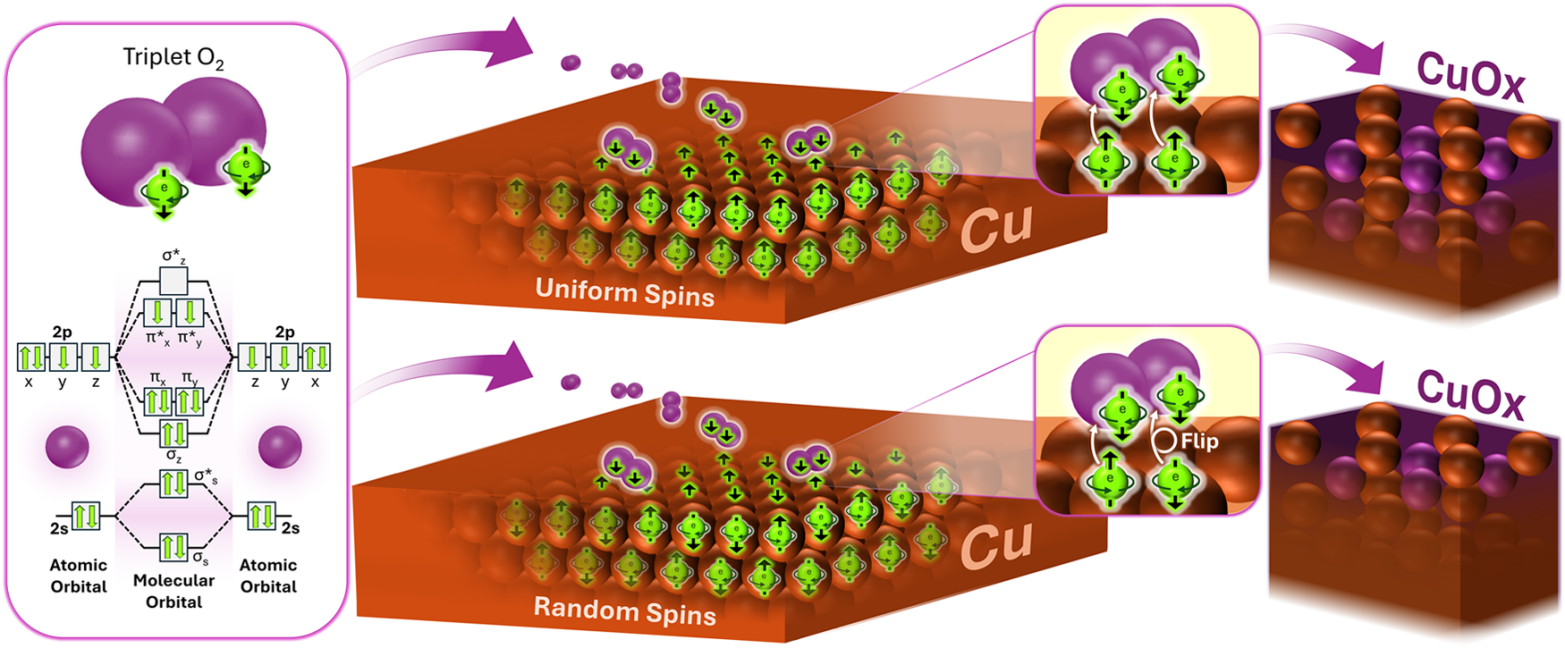
Spin-polarized Cu oxidation. Molecular orbital energy diagram of triplet oxygen (left). Illustration of the oxidation reaction on spin-polarized (top) and spin-unpolarized (bottom) copper, including the spin flip necessary during electron transfer in the case of unparallel surface spins, hypothesized to influence the rate and extent of oxidation.

To attain a broad survey of spin polarization involvement in Cu oxidation, we designed a series of experiments comparing the oxidation behavior of Cu films under varying spin conditions. Relying on the spin-polarizable properties of copper, we induce varying degrees of spin polarization on its surface through proximity to ferromagnetic nanostructures.^7,22,23^ By employing patterned ferromagnetic substrates and controlling the thicknesses of the gold capping layers, we created regions with different spin environments on the same sample. Various surface and bulk characterization techniques, probing the topographic, electrical, and magnetic sample properties, were applied in conjunction to monitor the oxidation of the copper films over time. These methods allow us to probe the influence of spin polarization on the oxidation process in a material system that is otherwise chemically uniform.

In all experiments, we find that differences in the spin polarization of the copper influence its oxidation process. These experimental results offer insight into the oxidation mechanism of copper and suggest a way to control passivation by aligning magnetic domains using chiral molecules.^20^

## 2 Results

In this work, we use three sample architectures defined below designed to possess regions of differing spin polarization. Our central hypothesis is that spin-polarized electrons, introduced via an underlying ferromagnetic surface, may influence the rate or mechanism of copper oxidation. To monitor these effects, we rely on material contrasts between metallic Cu and Cu oxide, such as differences in lattice constants, work function, and dielectric properties.

The primary characterization methods used are atomic force microscopy (AFM) and Kelvin probe force microscopy (KPFM), with complementary measurements conducted via ellipsometry and polar magneto-optic Kerr effect (P-MOKE). These techniques, in particular when applied in a correlated manner, allow us to assess the surface topography along with electronic and magnetic properties, tracking oxidation over time scales of minutes to weeks.

### 2.1 Grid Sample Architecture

Our initial experimental setup involved a grid pattern of perpendicularly aligned strips of 30 nm thick ferromagnetic (FM) Ni and 20 nm thick Cu films on a silicon wafer (Figure 2a). The Ni was capped by a 7 nm Au protective layer. The AFM topography measurements of the copper step thickness over time allowed for the observation of oxidation trends of copper on the two different surfaces, Si and Au/Ni. This type of topographical assessment is possible due to the increase in lattice constant when transitioning from metallic Cu to its two main oxides noted above.^7,24^ Measurements were conducted in the presence of an external magnetic field of 50 mT applied perpendicular to the surface to induce a uniform spin alignment in the FM layer.

**Figure 2:**
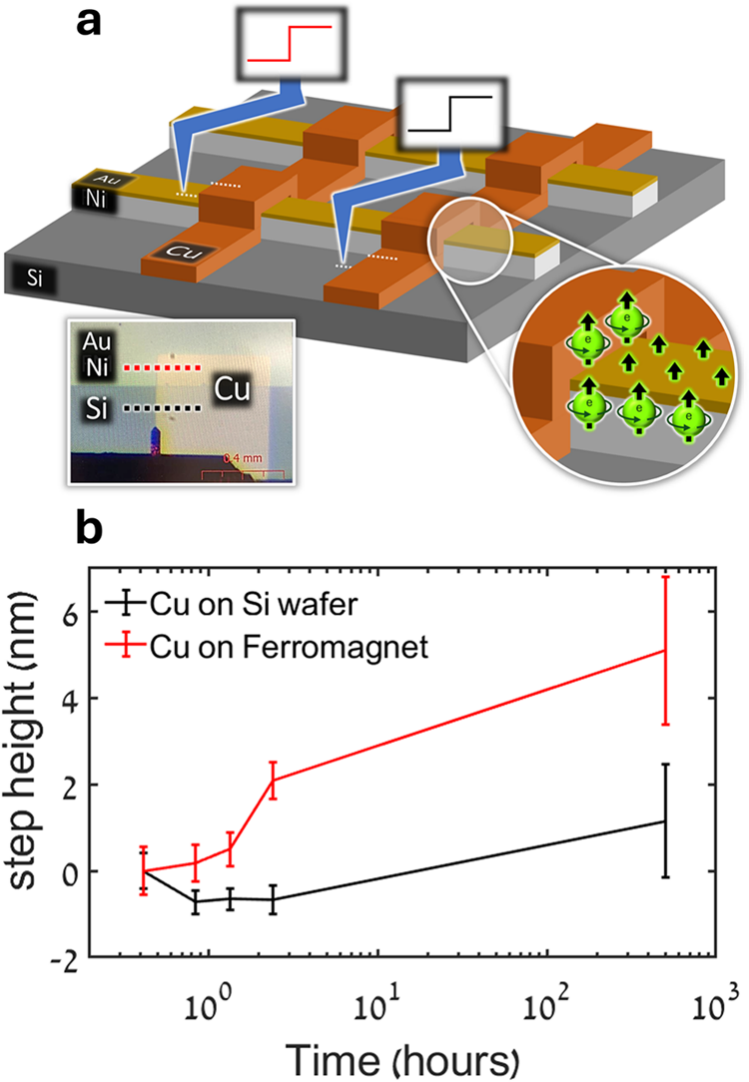
Grid sample. (a) Illustration and magnified optical image of the grid sample architecture showing Cu strips partly covering non-spin-polarized (Si) and spin-polarized (Au/Ni) regions. Dotted lines represent the AFM scanning profiles on the different regions of the sample. (b) Step thickness of Cu film deposited on either the bare silicon wafer or the Au/Ni ferromagnetic film, as a function of time, relative to the corresponding first measured thickness. Presented values are averages calculated from up to 256 scan lines per measurement, and the error bars represent corresponding standard deviations.

Figure 2b shows the thickness of the Cu layer as a function of time, for Cu undergoing oxidation atop the spin-polarized ferromagnetic layer or directly on the non-spin-polarized bare silicon. The values given are relative to the first measurement due to initially different step heights caused by the different mechanical and topographical properties of the substrates. The step profiles were obtained from 256 scan lines across a 5 × 5 um^2^ area on the step. We note an unexpected initial height decrease in the Cu film on Si, an issue we address below. From the overall increase in step height with time, it is evident that the Cu is oxidized on both substrates, but clearly at a faster rate when it is deposited on the Ni FM film than on the Si wafer. The oxidation rates approximated by the initial short-time data (under 3 h) are 1.3 and 0.03 nm/hour for the Ni and Si substrates, respectively, as demonstrated in Supporting Information Figure S1. Time dependences of the average step profiles for the two conditions are presented in Figure S2.

While these results offer an indication of the spin-dependent nature of Cu oxidation, the heterogeneous architecture of this sample type does not provide definitive information on the origin of the observed differences. While offering a comparison of Cu oxidation on top of magnetized (spin-polarized) Ni and nonmagnetic (nonpolarized) regions within the same sample, this design also introduces inherent, non-magnetic-related differences between the substrates. These differences, including roughness, lattice matching, and surface chemistry, may have structural influences on the Cu layer grown on them, resulting in uncertainty about the governing factors at the base of our observations.

### 2.2 Layered Cu/FM Films Architectures

To address the limitations of the initial setup, we developed another sample architecture, composed entirely of ferromagnetic substrates and possessing uniform surface chemistry. In this design, Cu films (of different patterns) were deposited on top of a gold layer covering a ferromagnetic cobalt (Co) film. The Au acts as a protective layer preventing Co oxidation, and the dependence of the Cu oxidation rate on Au thickness was monitored. Thicker Au layers act as strong spin barriers, impeding spin polarization from reaching the Cu, while thinner Au layers allow for better transmission of spin polarization from the underlying Co.

By having a uniform epitaxial Cu/Au/Co nanostructure across the entire substrate and varying only the Au capping thickness, we effectively eliminate the structural and chemical substrate differences. This is limiting the experimental variable to spin effects only, theoretically avoiding preoxidation structural differences of the Cu film. An additional important feature of the refined sample architecture is a ferromagnetic Co layer possessing strong Perpendicular Magnetic Anisotropy (PMA). This allows for samples to maintain an out-of-plane magnetization orientation and be measured in the absence of an external magnetic field, resulting in higher spin polarization than in the Ni. The long-term stability of the magnetization in the Co has been characterized previously using time-dependent polar MOKE measurements that showed no measurable changes in the hysteresis loop shape or the coercivity over time scales of months to even years, indicating a stable magnetic configuration over experimental time scales relevant to this work. Two design variants were implemented with this strategy: one, with a sharp boundary between thin and thick Au regions (Junction Samples), and the other, with a gradient in Au thickness across a single sample (Au Wedge Samples).

### 2.3 Junction Samples

For a clear comparison between regions differing in the degree of spin polarization, Au-capped (5 nm thick) FM cobalt (Co) substrates (1.5 nm thick) were partly covered by an additional 20 nm layer of Au, forming a step-junction. Cu films were then evaporated onto these substrates, and their oxidation at the two sides of the junction (step) was monitored by using combined topographic (AFM) and electrical (KPFM) measurements. The architecture of a junction sample is presented in Figure 3a, illustrating the four distinct regions of the substrates, the surface spin conditions induced in each, and the scan lines used. This design allows for scanning two-step profiles: between regions of Cu deposited on thin and thick Au films or directly on the Au of different thickness. Time-dependent trends of two-step profiles can then be monitored and compared for the oxidizing Cu versus inert Au. Figure 3b illustrates the possible observations to be expected in the measured parameters because of differences in the oxidation rates on top of thin and thick Au. As Au does not undergo oxidation, the bare Au–Au step serves as a reference measurement. We note that we do not have a quantitative measure of the degree of spin polarization penetrating through the Au film as a function of its thickness, but it was reported previously to decrease with thickness, and consequently, also the spin polarization at the Cu surface will be reduced.^25^

**Figure 3:**
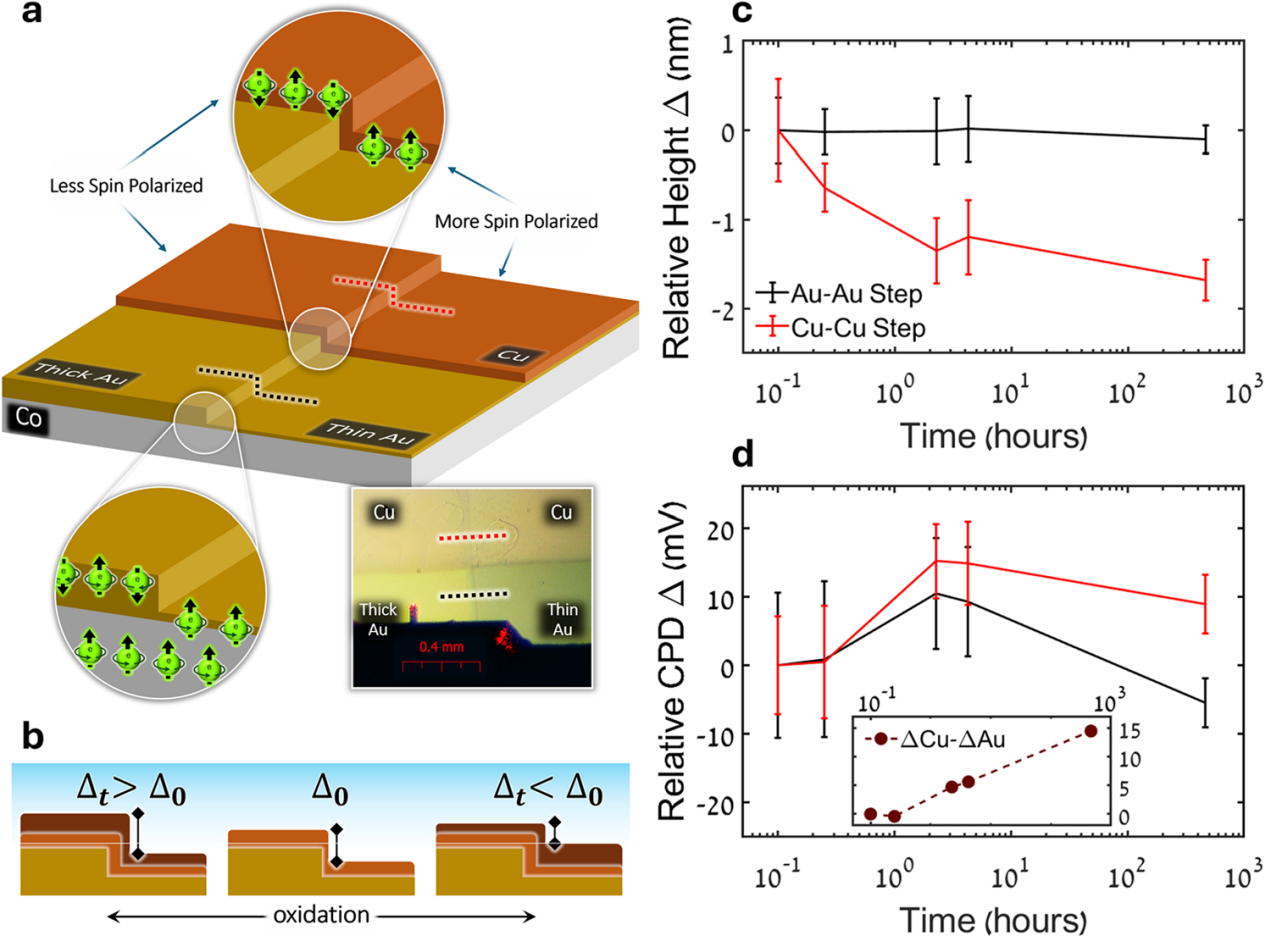
Junction sample. (a) Illustration and magnified optical image of the junction sample architecture, depicting the more spin-polarized and less-spin-polarized regions of the sample, controlled through the thickness of the Au capping layer atop the Co ferromagnet layer. Dotted lines represent the AFM scanning profiles on the different regions of the sample. (b) Illustration of possible changes in the Cu–Cu step height or CPD over time through oxidation, implying their expected effect on the various calculated Δ values. Dark brown layers represent copper oxide forming atop the Cu layer (light brown) and Au (yellow) layers. (c) Average AFM-acquired Cu–Cu and Au–Au step heights as a function of time relative to the first measured time point. (d) Average Cu–Cu and Au–Au step CPD values, in relation to the KPFM tip, as a function of time relative to the first measured time point. (inset) ΔCPD values achieved after subtraction of underlying changes in Au (ΔAu) values from the changes in CPD of Cu (ΔCu). Presented values are averages calculated from up to 256 scan lines per measurement, and the error bars represent corresponding standard deviations.

Figure 3c displays the height of the Cu–Cu step (Δ_t_) as a function of time, relative to the first time point (Δ_0_), measured using a topographic AFM scan. It can be seen (red line) that the height Δ between the Cu regions on thin and thick Au decreases over time, yielding negative Δ_t_ – Δ_0_ values. This implies that the oxidation of the Cu layer over the thin Au film occurs faster than that on the thick Au film, in accord with the expected degree of Au-surface spin polarization. The reference height Δ between thin and thick Au films (black) shows no significant change over time, as expected. Average topography profiles across the Cu–Cu and Au–Au steps for different times are presented in Supporting Information Figure S3.

The contact potential difference (CPD) was simultaneously measured by using KPFM with a Pt-coated AFM tip. Similarly to the topography measurements, the difference between the CPD for the Cu–Cu step (ΔCPD), measured above thick and thin Au layers, is presented relative to the first time point, (ΔCPD)_t_ – (ΔCPD)_0_. The evolution of this value is presented in Figure 3d (red line), showing first an increase and then a decrease over time. The corresponding data measured on the reference Au–Au step display a similar behavior (black), despite the ΔCPD of Au being expected to remain relatively constant over time. This behavior is most likely due to fluctuations in ambient conditions, such as temperature and humidity over time, which are known to affect measured CPD values.^26,27^ To account for these fluctuations and follow changes occurring solely in the Cu film, we subtract the CPD values of the reference Au–Au step from the Cu–Cu step, as plotted in the inset of the figure. Here, we observe an opposite trend in ΔCPD over time compared to the height Δ, with the difference between the CPD of Cu on thin and thick Au areas increasing over time and yielding positive (ΔCPD)_t_ – (ΔCPD)_0_ values. These results complement and further validate the height measurements. Since the work function of Cu increases as it is oxidized to either Cu_2_O or CuO, when calculating the CPD values compared to the Pt-coated Si tip, they are expected to decrease with oxidation.^28,29^ This would mean that an increase in the ΔCPD between Cu on thin and thick Au areas over time implies that oxidation is progressing faster on the thin Au. These trends agree with the grid sample measurements. It is important to stress that the measurements are conducted in the absence of any external magnetic field. This implies that the effect we show is not a simple dynamic one, induced by external fields, but instead is related directly to the surface spins.

### 2.4 Wedge Samples

An additional sample design used to control the spin conditions during Cu oxidation utilizes a wedge architecture. A wedge sample comprises a uniform 1.5 nm thick Co layer covered by a wedge Au film with thickness varying from 2 to 15 nm over a length of 10 mm. As before, the thickness of the Au layer, acting as a spin barrier, can dictate the extent of spin-polarized conditions at each location on the surface. While all capping thicknesses are within the range of reported spin diffusion lengths in Au, it has been shown that changes in spin coherence are induced by even small variations of the Au thickness atop a FM,^25,30,31^ To take advantage of the unique wedge sample properties, Cu strips were evaporated at different positions along it, exposing them to varying spin polarization conditions, offering further insight into the oxidation process. Although the exact dependence of the Cu surface spin polarization on the Au thickness is not known quantitatively, the wedge geometry enables systematic exploration across a continuous range of spin polarization conditions.

Figure 4a illustrates the wedge sample design, including three measured 15 nm thick Cu strips, induced with different degrees of surface spin polarization depending on the underlying Au thickness. Cu film at strip (position) 1, situated approximately over 4 nm thick Au capping is expected to be the most spin-polarized. Cu films at strips (positions) 2 and 3, situated over approximately 7 and 10 nm thick Au, respectively, are expected to be gradually less spin polarized. The step heights, averaged over all uncropped scan lines and relative to the first measurement (Δ_t_ – Δ_0_), are presented in Figure 4b for the three positions of the Cu strips. Evidently, the relative increase in step height, and therefore the degree of oxidation, decreases from position 1 to 3. This supports the hypothesis that Cu oxidation progresses faster under higher out-of-plane spin-polarized conditions, as seen in previous measurements. Like in the grid sample, here too, we observe an apparent decrease in height compared to the first measurement under the two less polarized conditions. The origin of this counterintuitive behavior is not fully understood. As expanded upon in the Discussion section, it may arise from measurement- or morphology-related effects during the early stages of oxidation. Importantly, since our analysis focuses on comparing the overall temporal trends and relative oxidation rates under different spin polarization conditions, this early-stage anomaly does not affect the main conclusions. The average height profiles for all three positions of Cu strips, for different measurement times, are shown in Supporting Information Figure S4a.

**Figure 4:**
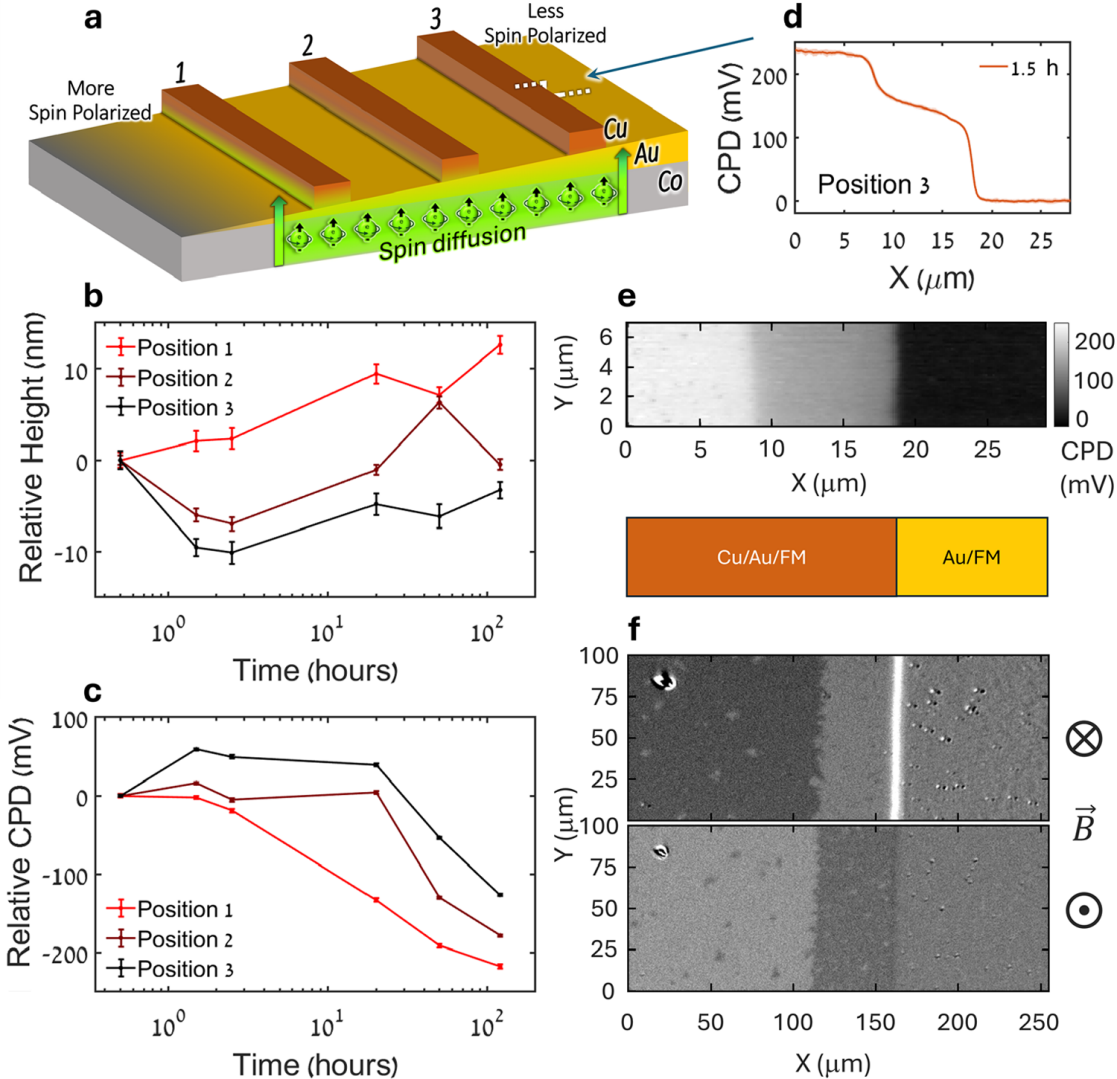
Wedge Sample. (a) Illustration of the wedge sample architecture, with Cu strips situated atop varying thicknesses of the Au capping layer, and the expected changing spin conditions along the sample’s length. (b) Step height as a function of time, relative to the first time point, for the three strips (positions), numbered according to the illustration in (a). (c) Au-subtracted CPD values as a function of time, relative to the first time point, for the Cu strip edge at the three measured strips (position numbers correspond to the scheme in (a)). Presented values in (b) and (c) are averages calculated from up to 256 scan lines per measurement, and the error bars represent corresponding standard deviations. (d) Representative two-step CPD profile, measured 1.5 h after exposure along the line illustrated in (a) at strip (position) 3, displaying a clear shoulder feature. (e) KPFM scan map of strip 3 after 1.5 h exposure, from which profile (d) was extracted, along with a schematic bar indicating which region consisted of a Cu strip (Cu/Au/Co) and which has only the bare underlayer (Au/Co). (f) P-MOKE images of Cu strip at position 2, under oppositely oriented out-of-plane magnetic fields as indicated, 5 weeks after exposure. The regions in the images adhere, but at a different scale, to the schematic bar provided in (e).

Figure 4c presents the CPD values of Cu, averaged over the scan area and relative to the first measurement, for the three measured Cu strips over time. The values are calculated after background subtraction of the measured CPD values of Au at the edge of the step (similar to Figure 3d, inset). A general trend of CPD decrease can be seen over time, with a faster decrease as conditions become more spin-polarized. This decrease, as mentioned above, represents an increase in work function, attributed to the oxidation of the Cu, implying, again, that the process is expedited under spin-polarized conditions. The average CPD profiles for all three strips at different times are plotted in Figure S4b.

Interestingly, some of the measured CPD profiles displayed a two-step feature, revealing a shoulder region at the edge of the Cu strip. One such profile, measured approximately 1.5 h after exposure at Cu strip (position) 3, is shown in Figure 4d. The shoulder (lower step) displays a CPD value intermediate to those of the center region of the Cu strip and the Au underlayer, meaning a higher work function compared to the interior of the Cu strip. The extent of the shoulder penetrating into the 500 μm wide Cu strip is approximately 10 μm from the edge, as seen also in the CPD map presented in Figure 4e. The two-step heights are plotted as a function of time in Figure S5, revealing that the edge feature does not persist within the measured scan range throughout the entire measured time. By the second day of measurement, only one step is detectable within the 40 × 40 μm scanned region. The plot also suggests that the edge feature appears and disappears earlier under more spin-polarized conditions compared to less polarized ones. A similar two-step feature is also present in P-MOKE microscopy images, as shown in Figure 4f for Cu strip 2. These images, taken more than a month after exposure, reveal an edge region of the oxidized Cu strip that induces a different magnetic behavior of the Co beneath, compared to the Co under the middle of the strip and to uncovered areas, as discussed below. P-MOKE microscopy relies on the polarization of light being rotated when it reflects off a magnetized surface. It utilizes carefully arranged cross-polarizers to create image contrasts based on differences in the orientation of magnetization at different regions in the visible section of the sample. By applying a varying external magnetic field and following changes in brightness, it is possible to compare the magnetic properties of chosen areas of the image. The two snapshots shown were taken for the opposite directions of an out-of-plane magnetic field sweep, at two subsaturation fields of about 12 and −12 mT. They capture a difference in the coercive field of the edge region compared to the interior of the Cu strip. The extent of the shoulder in this case is much larger than the one seen by KPFM, on the first day of exposure, and is about 50 μm deep. Figure S6 shows MOKE images of edge regions at Cu strips (positions) 1 and 2, and a later obtained hysteresis loop measured at position 2 can be seen in Figure S7.

### 2.5 Ellipsometry

To further investigate the Cu oxidation process, ellipsometry measurements were conducted using a full-Cu-coverage version of the junction sample architecture. Ellipsometry analyzes variations in light polarization upon reflection from a sample at oblique angles of incidence, providing detailed information about the sample’s properties and structure with atomic-level accuracy. It is highly sensitive to changes in the complex refractive index as well as to variations in the composition and thickness of the measured layers. In the case of Cu oxidation, the growth of an oxide layer, with a changing thickness and optical properties, alters the optical response of the structure, enabling ellipsometry to monitor the progression of the oxide film. Importantly, ellipsometry can be used to estimate the individual trends of Cu and its two main oxides. Figure 5a illustrates the accepted progression of Cu through its oxidation states over time, while Figure 5b shows the sample architecture used in the ellipsometry measurements. Due to the complex layered structure of the substrate and the lack of reliable information for some layers, separate ellipsometry measurements were conducted on the structure prior to the start of the oxidation process. These data were then used in the modeling to obtain the best representation of the structure, enabling the isolation of the response arising from variations in the growing oxide layer and thereby optimizing the accuracy and sensitivity of the results (see the Experimental Section for details). The obtained results, plotted in Figure 5c, show the total oxide growth trends for copper on both thin and thick Au layers (red and black, respectively), with a slightly faster growth observed on the thin Au. While this slight difference in trends is not as pronounced as in the other measurements, the analysis of the oxide composition shows the growth of Cu_2_O and CuO over both thin and thick Au regions. It can be clearly seen that the stronger spin-polarized conditions (thin Au) favor an earlier transition to CuO, while the weaker spin-polarized conditions (thick Au) delay it and sustain the Cu_2_O state for a longer period. It should be noted that the overall increase in thickness, as obtained from ellipsometry measurements, is smaller than that in the other methods and samples. This is possibly due to the favorable environmental conditions in which the ellipsometry samples were stored and measured under the controlled and stable temperature and humidity of a clean room facility. Supporting Information Figure S8 presents the different oxide contributions under each spin-strength condition as well as the trend of metallic Cu over time, showing a marked decrease in Cu thickness under the more spin-polarized conditions.

**Figure 5:**
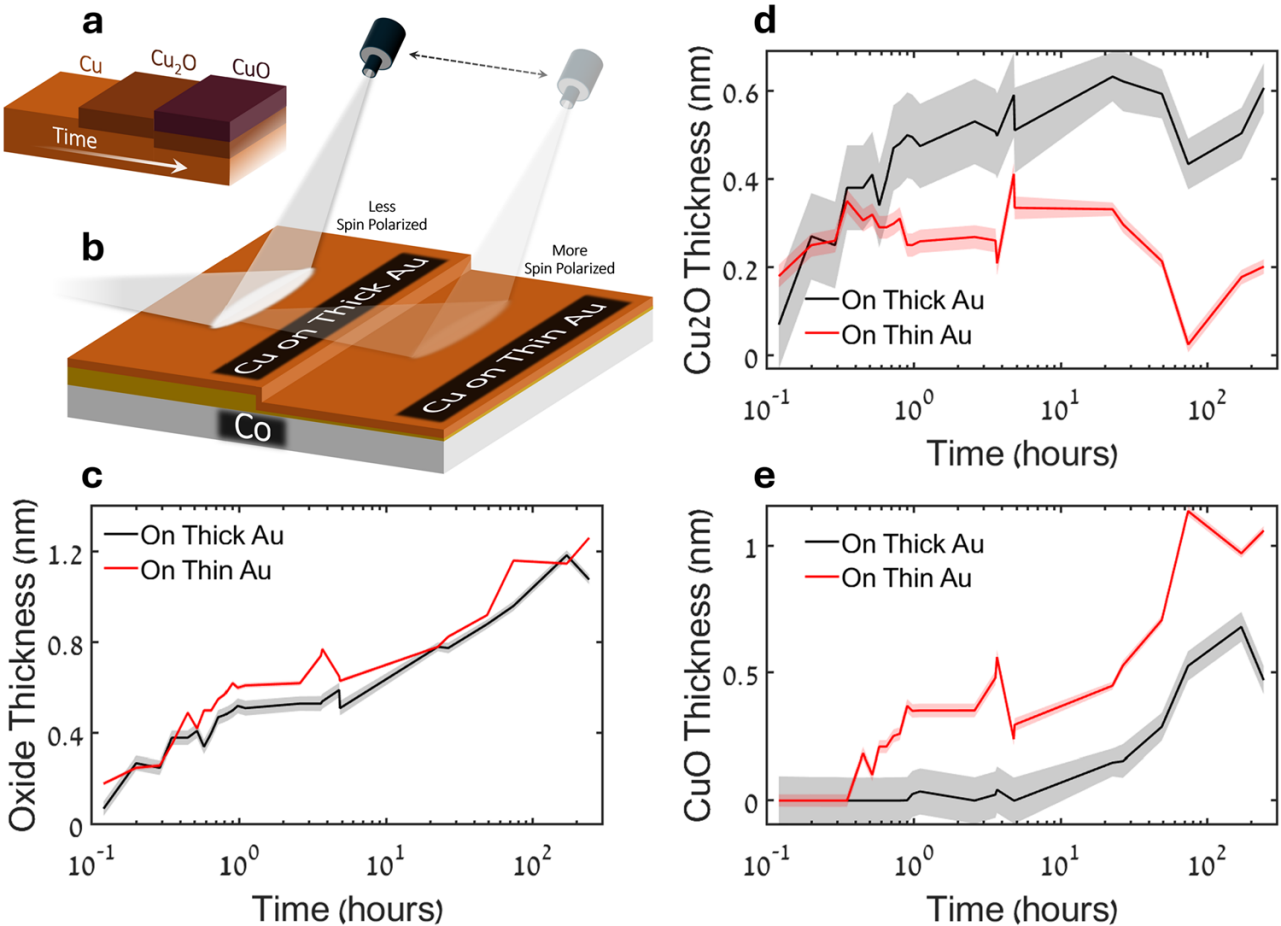
Ellipsometry. (a) Illustration of the evolution of copper over time, progressing from its first oxidation state (Cu^+^) in Cu_2_O to its second one (Cu^2+^) in CuO. (b) Illustration of the sample architecture used for ellipsometry measurements: Cu/Au layers of two different thicknesses on Co. (c) Total oxide thickness as a function of time for Cu deposited on either a thin or thick Au layer, corresponding to strongly spin-polarized and weakly spin-polarized conditions, based on ellipsometry measurements. The values are obtained using a mixing model of the two common oxides. (d) Cu_2_O thickness over time under the two spin polarization conditions, as derived from the oxide ratio obtained via the mixing model. (e) Similarly, CuO thickness over time under the two spin polarization conditions. Shaded areas represent the uncertainties in the extracted parameters.

## 3 Discussion

While the nature of this work is an initial survey, focusing on specific materials, it does provide insight into potentially fundamental factors in metal oxidation. In this section, we discuss the main observations and conclusions derived from the presented experiments and from the experience of performing them, with the aim of laying the path for future in-depth work in this field. One possible intriguing future extension of our work is comparing the oxidation rates using magnetic fields of different strengths, close to the coercive field and above the saturation field. Future experiments are aimed at quantifiably extracting oxidation rates under tightly controlled environmental conditions and quantifiably assessing the degree of spin polarization on the Cu and Au surfaces, using, e.g., magnetic force microscopy.

The experimental data collected here consistently indicate that spin polarization at the copper surface influences the oxidation kinetics. All measurements for all sample architectures display a clear difference in Cu oxidation trends under the varying surface spin polarization conditions, dictated by the thickness of the underlying Au film separating Cu from the Ni or Co films. Although we cannot quantify at this stage the exact value of spin polarization, the recurring observation from our various samples and measurements, showing higher oxidation rates for thinner Au layers, provides strong support for the trend of spin polarization, promoting oxidation. This is concluded from a faster, and more substantial, increase in film height and decrease in CPD, as seen in the AFM and KPFM measurements, respectively. Further insight is gained through ellipsometry analysis, showing that CuO formation is particularly favored under uniform surface spin conditions. Our measurements also suggest that the kinetics is nonlinear, except for a short initial stage, but the exact kinetics model cannot be determined at this stage. Additionally, an interesting edge effect appears to accompany the oxidation of the measured Cu strips with clear shoulder regions seen in both KPFM and P-MOKE measurements of the wedge samples. We interpret this edge effect as a general feature of copper oxidation, not inherently related to the degree of substrate spin polarization, reflecting the tendency for the process to initiate at step edges and defects.^32^ However, surface spin polarization enhances the rate of the entire process.

The main finding here of Cu oxidation being accelerated due to surface spin polarization alone should not be confused with dynamic effects known to be induced by magnetic fields. At the quantum-mechanical level, these results are consistent with the role of spin alignment in O_2_ redox chemistry.^19,33^ Molecular oxygen is a triplet (*S* = 1) in its ground state, so electron transfer involving O_2_ must obey spin selection rules favoring spin polarization. This was demonstrated in the context of O_2_ dissociation on Al(111) surfaces, suggesting unaccounted spin-selection restrictions make triplet O_2_ dissociation highly nonadiabatic.^14^ By analogy, a spin-polarized Cu surface, in which metal electron spins are mostly aligned, should facilitate spin exchange with adsorbed O_2_ and effectively lower the spin barrier for electron transfer. In other words, when the metallic Cu electrons are uniformly arranged as spin-up or spin-down, transferring an electron into O_2_’s parallel-spin π* orbital can proceed with less spin mismatch.

This picture is supported by observations of electrocatalysis by spin-polarized metal centers preferentially promoting triplet O_2_ formation via quantum spin exchange.^15,18,19^ In our system, the proximity to the FM underlayer imposes different spin polarization conditions on the adjacent Cu layer so that the initial electron donation to O_2_ is, correspondingly, either more or less spin selective. This evidently yields a more rapid conversion of Cu to its oxides under the more spin-aligned conditions, fitting well with the original assumptions.

Based on this rationale, spin alignment is expected to be most impactful at the first stage of oxidation, from Cu to Cu_2_O, through better electron transfer between the spin-polarized metal surface and the triplet oxygen. However, as seen in the ellipsometry measurements, spin-polarized conditions seem to affect also the second oxidation process, from Cu_2_O to CuO. The analysis of these measurements implies that for the more uniform surface spin conditions (for a thin Au area), CuO formation occurs at an earlier stage compared to the case with random spin conditions. The persistence of surface spin polarization, following the first oxidation step, is not trivial considering mixed reports on the spin permeability/insulation of Cu_2_O.^34,35^ The involvement of spin alignment in Cu_2_O to CuO conversion, however, is perhaps less surprising considering the unique magnetic properties measured for this second oxidation state. Although Cu_2_O, similarly to Cu, has no unpaired electrons and is diamagnetic, CuO has unpaired electrons in the 3d orbitals of Cu^2+^ and is paramagnetic at room temperature or even antiferromagnetic at low temperatures.^34,36,37^ In conjunction with the ellipsometry results, we may assume that the induced spin alignment in this magnetically active phase promotes a faster second oxidation reaction. This, once again, is likely to be correlated with the triplet configuration of the incoming O_2_ molecules, this time with the added factor of a more naturally spin-aligned product in CuO.

There may well be another mechanism that can further enhance the oxidation rate on spin-polarized Cu surfaces in addition to lowering the intermediate energy barrier of the oxidation process. As the triplet O_2_ molecules are paramagnetic, they should be attracted to magnetized (spin polarized) surfaces significantly more than to an unpolarized surface. These two mechanisms should act in parallel, both increasing with surface spin polarization, and we could not decipher their relative contributions.

One surprising KPFM observation in the wedge sample is that of a prominent shoulder (two-step) feature at the edge of the Cu strip (Figure 4d). This feature, appearing only in the CPD and not in height (AFM topography) data, represents a markedly higher work function in the edge region of the Cu film compared to its center area. This two-step feature disappears from the scanned region over time as the lower CPD step (higher work function) moves deeper into the strip at the expense of the higher (lower work function) step. This process appears to become more rapid with increasing degrees of spin polarization (Figures S3b and S4). This increase in work function at the Cu strip edge, indicative of copper oxide formation, suggests a propagative pattern to the oxidation process, from the edge of the strip inward. The occurrence and behavior of this edge feature possibly reflect the progressing steps of Cu oxidation. As the initial oxidation of Cu occurs rapidly, the first appearance of the two-step feature must coincide with a markedly faster oxidation at the edge region. The inner step, displaying a higher CPD (lower work function), is likely associated with a low depth and degree of Cu oxidation, mostly composed of Cu_2_O and a persisting metallic Cu layer. The outer step, possessing a lower CPD (higher work function), can therefore be assumed to have a significantly higher depth and degree of Cu oxidation, including little to no metallic Cu. The low, and continuously decreasing, CPD of the outer step is also likely indicative of high, and increasing, ratios of CuO compared to Cu_2_O. Cu oxidation is known to be nucleated most easily at grain boundaries and sites of impurities and defects, and is also preferentially induced at certain facets of the Cu crystal structure, most notably Cu(110).^38–40^ Concurrently, thermally evaporated Cu films predominantly present the less reactive Cu(111) facet on their major exposed surface, and their oxidation has been found to be initiated mostly at steps and terraces.^41,42^ The oxidation shoulder is likely not detectable in later measurements, as it propagates inward from the edge and out of the scanning range of the AFM/KPFM. The faster propagation seen in the case of the more spin-polarized films supports the conclusion that Cu oxidation by O_2_ is expedited under these conditions. The lack of a shoulder feature in the topographic AFM scans probably results from the different sensitivity available when following the morphological or electronic changes accompanying the oxidation process.

While the propagating oxidation is not seen after the first day in the KPFM measurements due to the limited scan range, P-MOKE images (covering much larger length scales) taken several weeks after exposure show a similar feature penetrating the film much deeper into the film. This edge region, as seen in the magnetic hysteresis measurements (Figure S6) obtained on Cu strip position 2 after several months, is characterized by a higher coercive field than the inner region of the Cu film (approximately 22 vs 10 mT). As the visualized magnetization is of the underlying Co ferromagnetic layer and not of Cu itself, the observed difference likely reflects changes induced in it by the top layers. Owing to the paramagnetic properties of CuO, it is possible that regions rich in this oxidation phase would have a unique effect on the ferromagnetic layer compared to regions dominated by diamagnetic phases. However, mechanical strain is also known to induce changes in ferromagnetic properties and might also be a possible cause.^43^ As edge nucleation of Cu oxide is expected and oxidation involves physical expansion and rearrangement of the crystal structure, unique strains could easily be induced on adjacent materials. The center region of the film might be less oxidized, resulting in less stress induced on the FM substrate in that area.

Interestingly, in some of the topographic measurements, we see what appears to be an initial decrease in the step height with time during oxidation, under the less spin-polarized conditions, before the expected increase takes over. This behavior is observed in the grid sample (Figure 2) as well as in the wedge sample (Figure 4). This peculiar phenomenon is likely an artifact of the measurement, possibly caused by changes in the electric and mechanical force interactions between the tip and the sample, as metallic Cu is morphed into soft dielectric oxides. This type of behavior has been documented and utilized in other works, confidently correlating oxidation processes with adhesion and mechanical resistance changes, influencing phase contrast, and often obscuring AFM readouts.^44–46^ While tip–sample interactions might explain an initial decrease in step height as measured by AFM, they are expected to appear similarly in both spin-polarized and nonpolarized conditions. The reason it is not detected in the polarized case is possibly due to the increased rate of oxidation, where the oxide thickness compensates for the effect described above. The buckled shape of Cu as it is oxidized unevenly at different regions and facets could also result in a tilting and bending of the film, possibly reducing the measured height in some instances. An intriguing alternative explanation might be found in the different thermal expansion properties of Cu, Cu_2_O, and CuO. X-ray photoelectron spectroscopy measurements, presented in the Supporting Information, reveal the oxide compositions to be approximately 60% Cu_2_O and 40% CuO, confirming the presence of both oxide phases in our samples. Cu displays larger thermal expansion compared with its oxides, of which Cu_2_O shows the smallest and, in some cases, even negative thermal expansion (NTE).^47,48^ The involvement of this unique property, particularly under less spin-polarized conditions, would support the idea of a faster Cu_2_O to CuO transition under high spin-polarized conditions, as found by ellipsometry measurements. However, NTE in Cu_2_O was seen only at low temperatures (less than 250 K) and is not typically expected to be observable under the ambient and room temperature conditions of the experiments. While we cannot currently definitively prove these causes, and they warrant a more in-depth and systematic investigation of the observations for a clearer understanding of their veracity and origins, we believe they do not contradict the general trends and conclusions. In all cases, we observed an upturn and the overall behavior was an increase in film thickness with a faster rate under more spin-polarized conditions. Importantly, the time-dependent height evolution is only one of four parameters used to follow the oxidation process, applied on different sample configurations, all indicating that the oxidation rate and dynamics depend on surface spin polarization.

Our findings may have implications for practical processes involving copper surfaces. In catalysis, a preference for CuO over Cu_2_O could affect the reaction selectivity and rates. In spintronic devices, controlling oxide formation at interfaces may help stabilize their performance.

## 4 Conclusions

We find here, using topographic, electronic, magnetic, and optical measurements of spin-controlled multilayer hybrid films (Cu–Au ferromagnet), that spin polarization influences the process of Cu oxidation by triplet molecular oxygen. The results suggest a general increase in the rate of oxidation and a preference for the second oxidized state of copper, CuO, under spin-polarized conditions. This acceleration of oxidation under spin-aligned conditions falls in line with the hypothesis that spin exchange can lower the barrier for electron transfer into O_2_’s parallel-spin orbitals, with possible support of attraction of the paramagnetic O_2_ molecules to the spin-polarized surface.

Another interesting and important implication of our findings is that the triplet nature of molecular oxygen can slow spontaneous oxidation for many metals under normal conditions. Because O_2_ is in a triplet state, efficient electron transfer to form an oxide requires spin alignment, which is not always readily achieved. While our study focuses on copper, this spin-dependent oxidation mechanism could, in principle, be applied to other metals as well. The extent of the effect, however, will depend on the specific electronic and magnetic properties of each metal.

The evidence presented here supports the idea that spin is not merely a spectator in metallic surface redox chemistry and that surface spin chemistry should be differentiated from external magnetic field effects. Implications of this may range from revisiting the underlying factors governing the oxidation process itself to applying them in controlled induction or the prevention of metal oxidation in material science research and industry. Our results potentially pave the way for better manipulation of surface reactivity in catalysis, for improved spintronic design, and for general corrosion mitigation.^49^

## 5 Experimental Section

### 5.1 Grid Samples

Polished intrinsic Si wafers with native oxide were used as substrates for the grid samples. After dicing into 5 × 5 mm pieces and cleaning in boiling acetone, ethanol, and plasma asher, ferromagnetic strips of 30 nm thick Ni and 7 nm thick Au overlayer were prepared. 20 nm thick strips of Cu were then deposited on top. The Cu strips were deposited on the substrates by thermal evaporation through a patterned rigid mask. Following Cu deposition, the samples were kept under inert conditions in the ultralow O_2_, ultralow H_2_O, N_2_ environment of the glovebox until retrieved for measurement.

### 5.2 Junction Samples

Junction samples were prepared on 5 × 5 mm cobalt-based ferromagnetic substrates, showing perpendicular magnetic anisotropy (PMA). The substrates were fabricated by Prof. Lech Tomasz Baczewski of the Polish Academy of Sciences, Institute of Physics. These unique substrates consist of a meticulously designed sequence of layers deposited at different temperatures atop a (0001) Al_2_O_3_ (sapphire) substrate using the molecular beam epitaxy (MBE) method. The sequentially deposited epitaxial layers and their thicknesses are Pt (5 nm), Au (20 nm), Co (1.5 nm), Au (5 nm), yielding PMA and a coercive field of 150 Oe. The junction sample layers were prepared on the PMA substrates by thermal evaporation. First, a 20 nm film of Au was deposited through a rigid mask to cover one-half of the sample, then a 20 nm film of Cu was deposited to cover a 90° rotated half of the sample. A junction was thus created at approximately the center of the sample between its four different quarters, presenting exposed PMA substrate (thin Au), thick Au, Cu on thin Au, and Cu on thick Au. Samples were kept under inert glovebox conditions following the evaporation until extracted for measurement.

### 5.3 Wedge Samples

The preparation of wedge samples was also conducted on PMA substrates fabricated by Prof. Baczewski. However, these 5 × 10 mm slices possess an additional unique feature, with a thickness gradient in the capping Au layer deposited as a wedge. The gradient spans the whole length of the sample, going from 2 nm Au on one side to 15 nm on the other. 15 nm thick strips (0.5 × 3.5 mm) of Cu were then deposited by thermal evaporation at different positions along the Au wedge, using a patterned rigid mask. As with all sample types, these were kept in the glovebox until used.

### 5.4 Ellipsometry Samples

Samples prepared for use in the ellipsometry measurements were fabricated very similarly to the junction samples, with changes mostly in dimensions and Cu coverage. The substrates of these samples have the same layer structure as those used for the junction samples but are 10 × 10 mm in size and have a coercive field of 175 Oe. As with the junction samples, a 20 nm Au film was thermally evaporated on half of the substrates through a rigid mask. The 20 nm Cu film, however, was deposited over the entire area of the sample, covering both thin and thick Au regions completely. The larger sample size and full Cu coverage were chosen for compatibility with the spot size of the ellipsometer beam, which is approximately 3 × 9 mm.

### 5.5 Fabrication Instrumentation

With the exception of the MBE-grown PMA substrates provided by Professor Baczewski, all additional sample fabrication and preparation were conducted at the Hebrew University of Jerusalem. This was done in the clean room facilities of the Harvey M. Krueger Family Center for Nanoscience and Nanotechnology. Sample cleaning before every evaporation step included an O_2_ plasma treatment in a Diener Pico Plasma Asher, at 50% power for 10 min. All thermal evaporations were performed using a custom-made VST glovebox evaporator with a rotating sample holder, operated by Mr. Alexander Oginets. Prior to exposure, samples were stored and handled within a custom-made Mbraun glovebox system, where the O_2_ and H_2_O levels are maintained below 1 ppm.

### 5.6 AFM and KPFM Measurements

All atomic force microscopy measurements were conducted at ambient conditions using an NTEGRA (NT-MDT) AFM system, controlled by Nova software, with Pt-coated Si cantilevers (∼2 N/m force constant, ∼130 kHz resonance frequency). The quality of each tip was regularly monitored using a pristine Au reference measured under the same conditions, and tips were replaced immediately if any change in the reference CPD was detected. 40 × 40 μm scans of 256 × 256 pixels were made, and in some cases, cropped to exclude contaminated inhomogeneous regions. The processing and analysis of the obtained topographic scans included linear slope corrections (to account for an overall sample tilt) and manual step detection. For each strip and time of measurement, topographic step heights and CPD differences across the edges were calculated by averaging over areas of up to 200 μm^2^ (but typically less), extending more than 5 μm on either side of the assigned step region. Representative processed scan images and step calculation profiles for strip 2 on the wedge sample are shown in Figures S9 and S10.

### 5.7 Ellipsometry Measurements

Ellipsometry measurements were conducted using a J.A. Woollam Co. alpha-SE ellipsometer, in the 380–900 nm spectral range, at three incident angles of 60, 70, and 75°. Both thin and thick regions of a sample were measured prior to Cu evaporation and then periodically, following Cu exposure. Result analysis and interpretation were performed using J.A. Woollam CompleteEASE 6 software. To ensure optimized accuracy in isolating the oxide layer results in this complex multilayer ellipsometric analysis, we first measured the substrate alone and modeled its multiple layers on a wavelength-by-wavelength basis to determine their effective optical constants. We then measured the sample immediately after copper deposition and applied the same approach to the copper layer on the substrate, deriving its optical properties on a wavelength-by-wavelength basis at a fixed thickness of 20 nm (which aligned well with the literature values for copper). This method allowed us to obtain the most accurate representation of the multilayer structure beneath the oxide layer, which is the focus of our analysis. This is crucial, as inaccuracies in modeling the underlying layers would erroneously affect the fitting of the oxide layer properties and yield misleading results. When fitting the properties of the oxide layer, we employed a multi-sample analysis (MSA) for accuracy and consistency, simultaneously fitting 36 different ellipsometry data sets taken at various times after the start of the oxidation process. We used the copper layer thickness as a free-fit parameter to observe any progressive variation (if present) during the formation of the oxide layer. The oxide layer was modeled as a Bruggeman EMA (effective medium approximation) layer composed of a mixture of Cu_2_O and CuO. Finally, to extract the data of interest, the thickness of the copper layer, the total thickness of the oxide layer, and the percentage of CuO in the oxide layer were simultaneously fitted using the MSA (for thin and thick layer samples separately).

### 5.8 MOKE Imaging

Wedge Sample MOKE imaging was performed using a commercial Evico Magnetics GmbH magneto-optical Kerr microscope. A polar configuration was used, with an out-of-plane magnetic field generated by using electromagnets. For the optical imaging of magnetic samples, 20× commercial Zeiss objective lenses were used. For the MOKE imaging, first, a magnetic field was applied to saturate the sample. Then, the saturated image was subtracted from the optical image to enhance the magnetic image contrast. Initially, only imaging was performed, revealing the discussed magnetic edge effect, but no precise magnetic data was recorded. Unfortunately, when revisited for in-depth characterization and hysteresis measurements several months later, the sample appeared to have suffered some damage and measurements were limited to a small region. Hysteresis measurements were conducted in a range of −40 to 40 mT, in increments of approximately 1.35 mT.

## Supplementary Material



## Notes

Additional AFM and KPFM measurements of copper oxidation kinetics for the grid, junction, and wedge sample architectures; average height and CPD profiles as a function of oxidation time; MOKE images and magnetic hysteresis loops; ellipsometry analysis of Cu, Cu_2_O, and CuO thickness evolution; and XPS characterization of the copper oxidation states (PDF)
