# Supplementary material for: The Effects of Surface Spin Polarization on Copper Oxidation by Triplet Oxygen
Source: ACS Nano. 2026 Feb 11;20(7):6234–45. doi: 10.1021/acsnano.5c21063 (PMC12947736; doi:10.1021/acsnano.5c21063)

Supplementary Information

For:

The effects of surface spin polarization on copper oxidation by triplet oxygen

Avi Schneider,[1] Meital Ozeri,[1,2] Yael Kapon,[1] Ralfy Kenaz,[2] Vitaly Gutkin,[3] Shira Yochelis,[1] Lech Tomasz Baczewski,[4] Doron Azulay,[2,5] Oded Millo,[2] Yossi Paltiel[1]

[1] Applied Physics Department and Center for Nano-Science and Nano-Technology, The Hebrew University of Jerusalem, Jerusalem 9190401, Israel.

[2] Racah Institute of Physics and the Hebrew University Center for Nanoscience and Nanotechnology, The Hebrew University of Jerusalem, Jerusalem, 91904, Israel.

[3] The Center for Nanoscience and Nanotechnology, The Hebrew University of Jerusalem, Jerusalem 91904, Israel.

[4] Institute of Physics Polish Academy of Sciences, Al. Lotnikow 32/46, 02-668 Warszawa, Poland.

[5] Department of Physics, Azrieli College of Engineering, Jerusalem 9103501, Israel

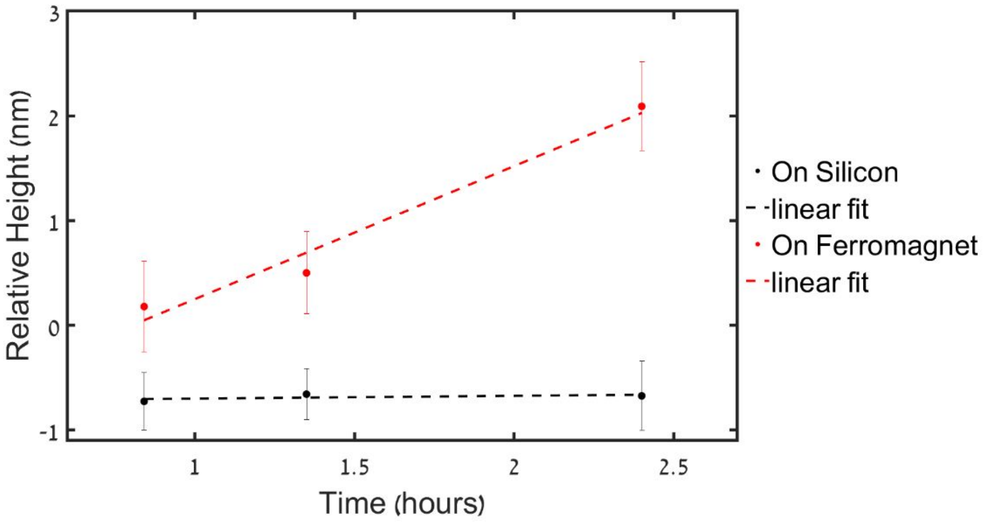

Figure S1 – Linear approximation of oxidation rate. *Step height of the grid sample taken at relatively short oxidation times in order to approximate the initial linear oxidation rate. The analysis yields a rate of about 1.3 vs 0.03 nm/hour for the spin polarized vs non-polarized copper strips.*

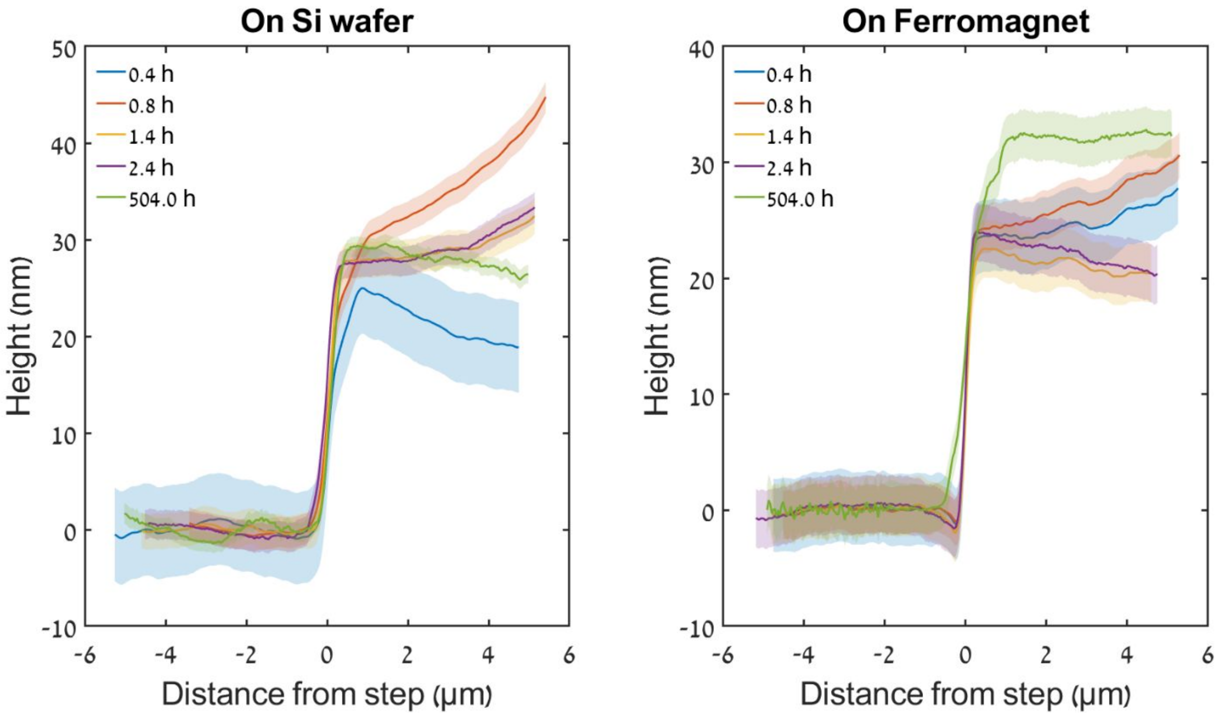

Figure S2 – Grid Sample average height profiles. *Average Cu step profiles, measured as a function of time (as indicated in the legends) by AFM, on either the Si (left panel) or Au/Ni (right panel) regions of the Grid Sample. Height values are measured with respect to Au or Si zero baseline for comparative analysis. The distance values are presented in a way to align all step profiles with one another and center them around the step position, x=0, with the Cu film to the right. The presented profiles are averages of up to 256 scan lines per measurement, and the shaded area represents the standard deviation around this average for each curve.*

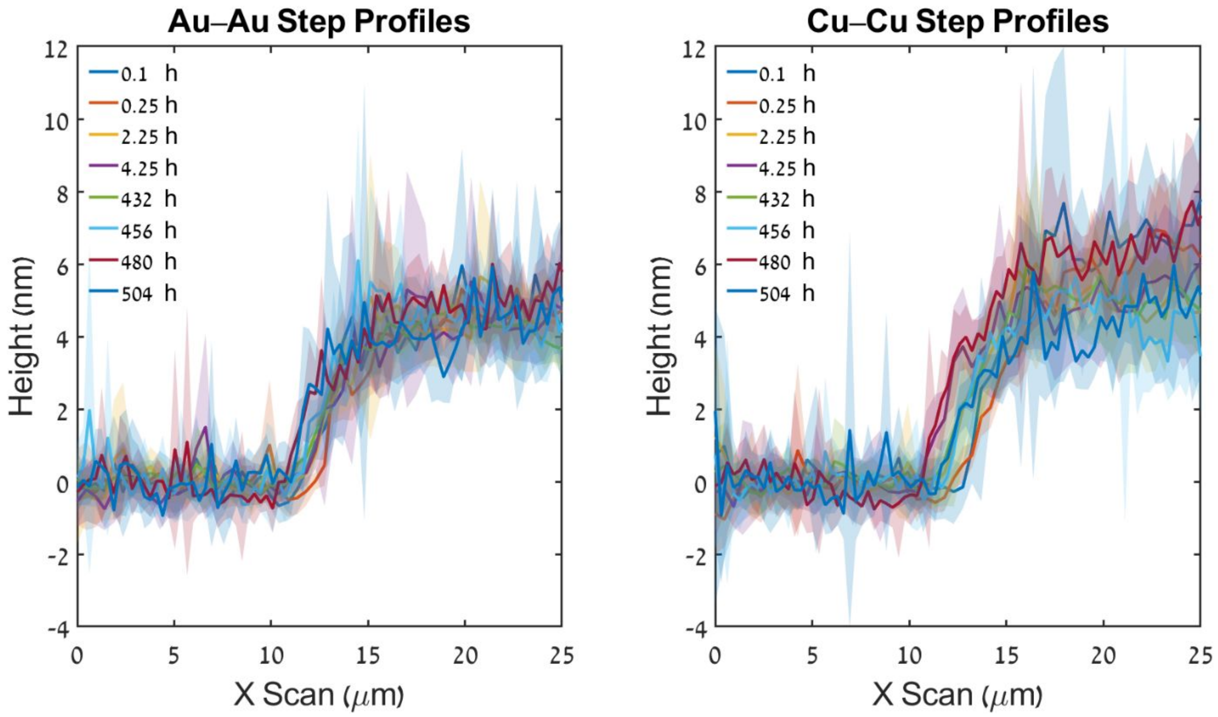

Figure S3 – Junction Sample average height profiles. *Average Au-Au (left) and Cu-Cu (right) step profiles, measured as a function of time (indicated in the legend) by topographic AFM, spanning a 10 μm² spatial region on either side of the step. Height values are plotted with respect to the lower side of the step for comparative analysis. The distance values are presented in a way to align all step profiles with one another and center them around the step position, x=0, with the thin Au-film side to the left. The presented profiles are averages of up to 256 scan lines per measurement, and the shaded area represents the standard deviation around this average for each curve.*

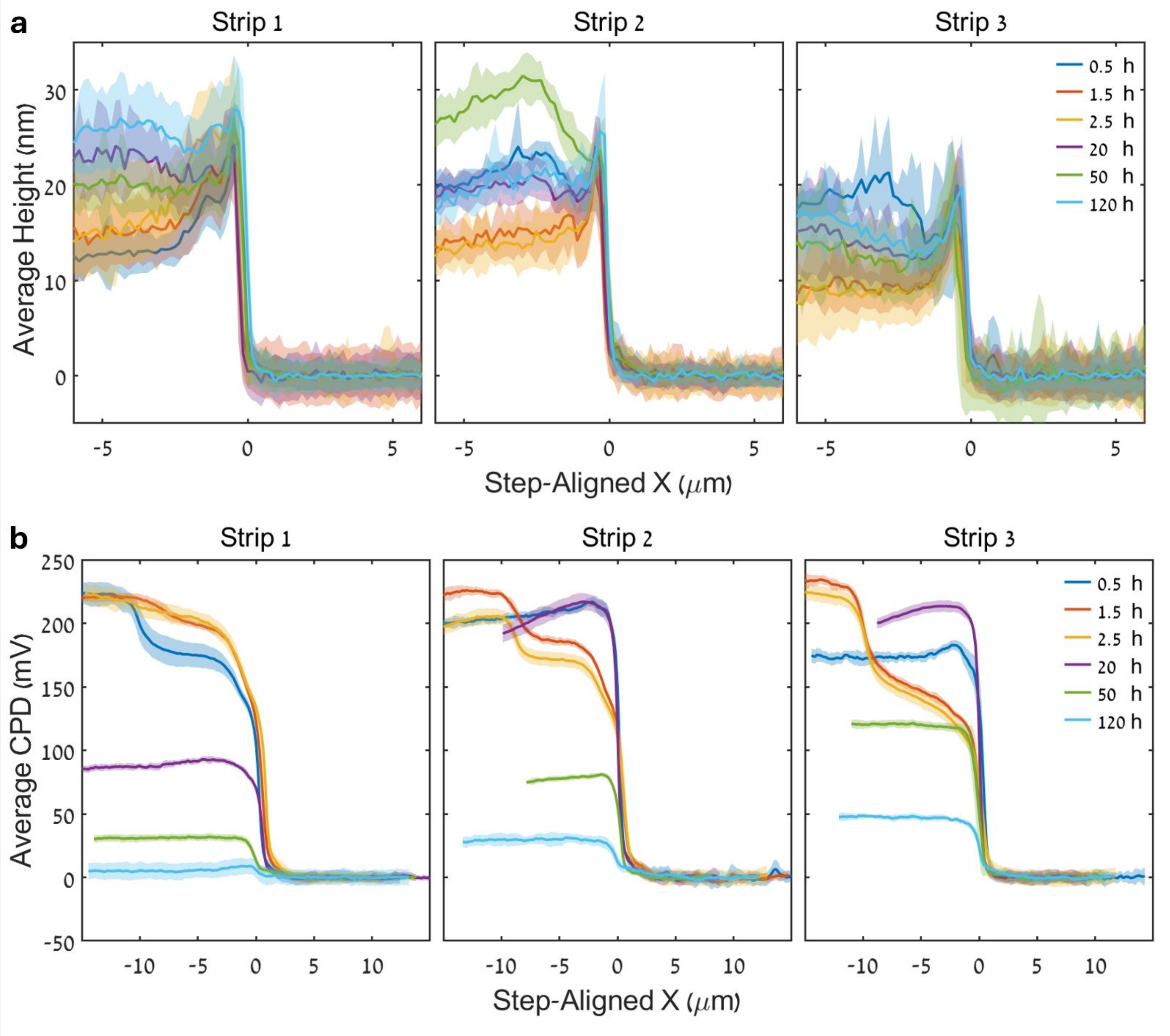

Figure S4 – Wedge Sample average height and CPD profiles. (a) *Average Cu step profiles, measured as a function of time (indicated in the legend) by AFM, at the three strips on the Wedge Sample. Height values are normalized to an Au zero baseline for comparative analysis.* ***(b)*** *Average CPD step profiles, measured as a function of time by KPFM, at the three strips on the Wedge Sample. CPD values are normalized to an Au zero baseline to isolate Cu oxidation trends from ones induced by changes in ambient experimental conditions. In all panels the distance values are presented in a way to align all step profiles with one another and center them around the step position, x=0, with the Cu film to the left and Au to the right. The presented profiles are averages of up to 256 scan lines per measurement, and the shaded area represents the standard deviation around this average for each curve.*

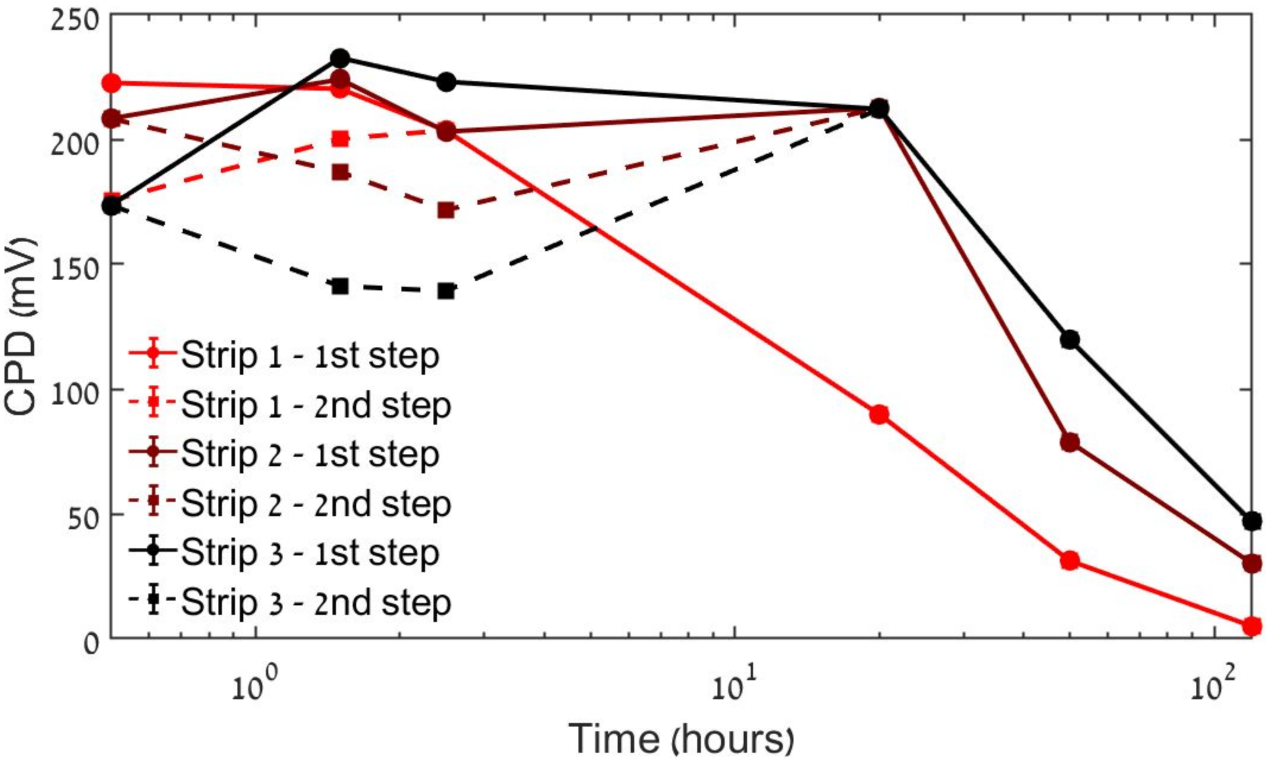

Figure S5 – Wedge Sample CPD first and second step evolution. *Average CPD values (normalized with respect to the Au CPD, as explained in the main text) for both the bulk (second step – bold line) and the shoulder (first-step – dashed line) of the Cu film, at the three Cu strips (color coded) on the Wedge Sample. The merging of the two lines for each strip indicates the shoulder disappearance from the scanning area.*

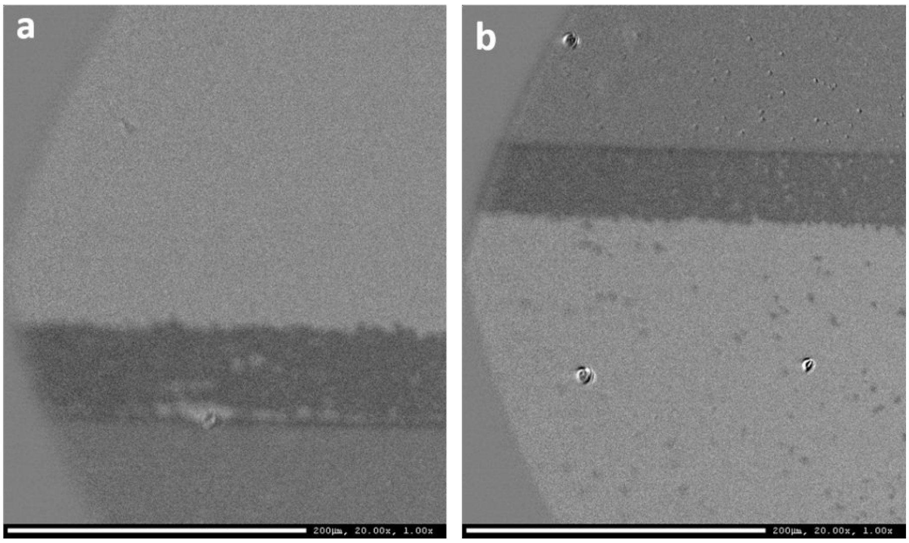

Figure S6 – MOKE images. *MOKE microscope images of the edge region at strips 1(a) and 2 (b) showing the unique magnetic behavior induced in this area compared to the adjacent regions further inward under the Cu strip and outward to the uncovered substrate. Scale bars are 200 μm.*

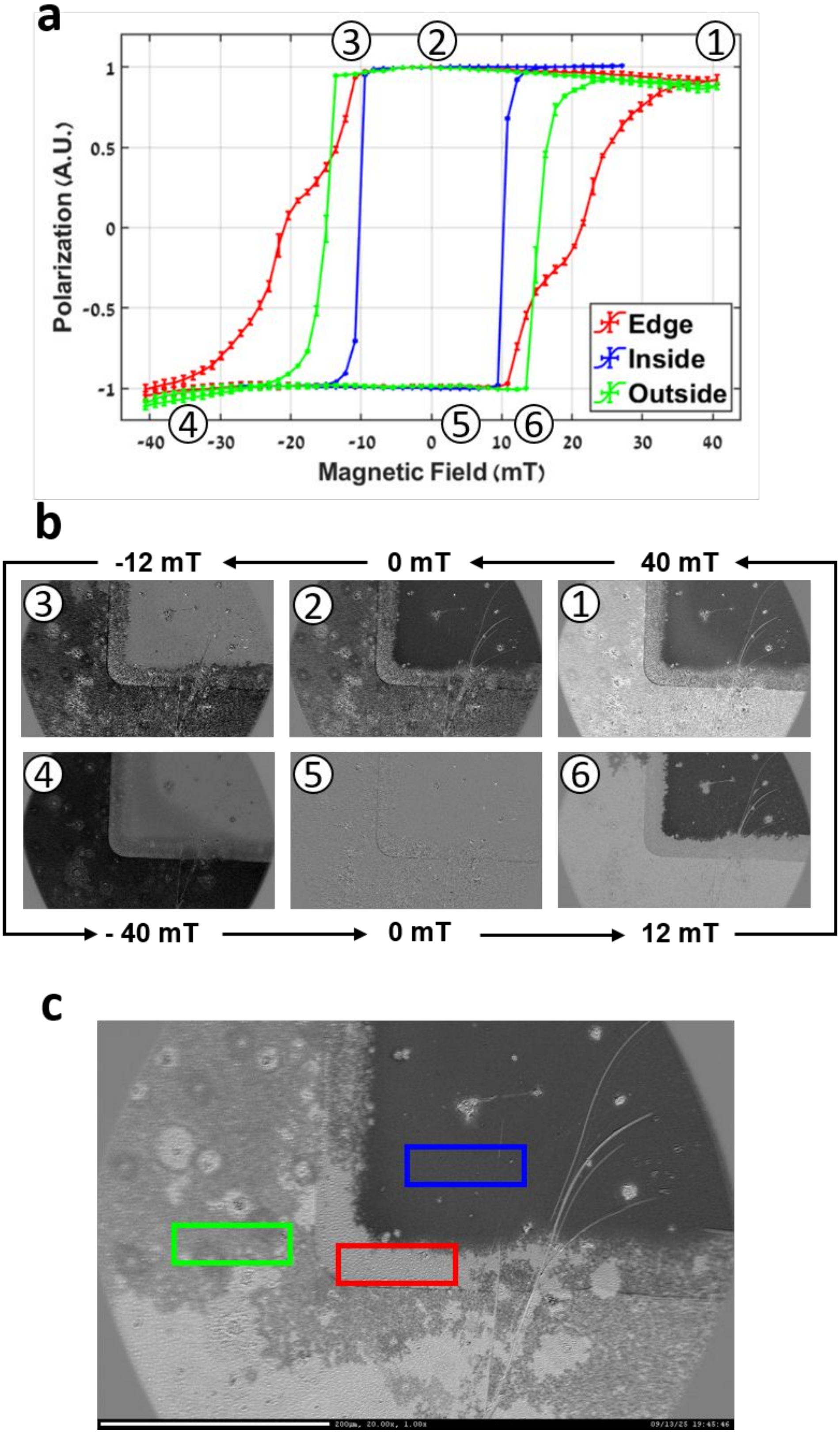

Figure S7 – Hysteresis loops. ***(a)*** *Magnetic hysteresis loops for three regions around the edge of Cu strip 2, obtained using MOKE microscopy.* ***(b)*** *Representative MOKE images taken at various out-of-plane applied magnetic fields (as indicated) throughout*

the magnetic-field sweep. ***(c)*** *Locations of the analyzed regions around the strip edge, with colors corresponding to (a).*

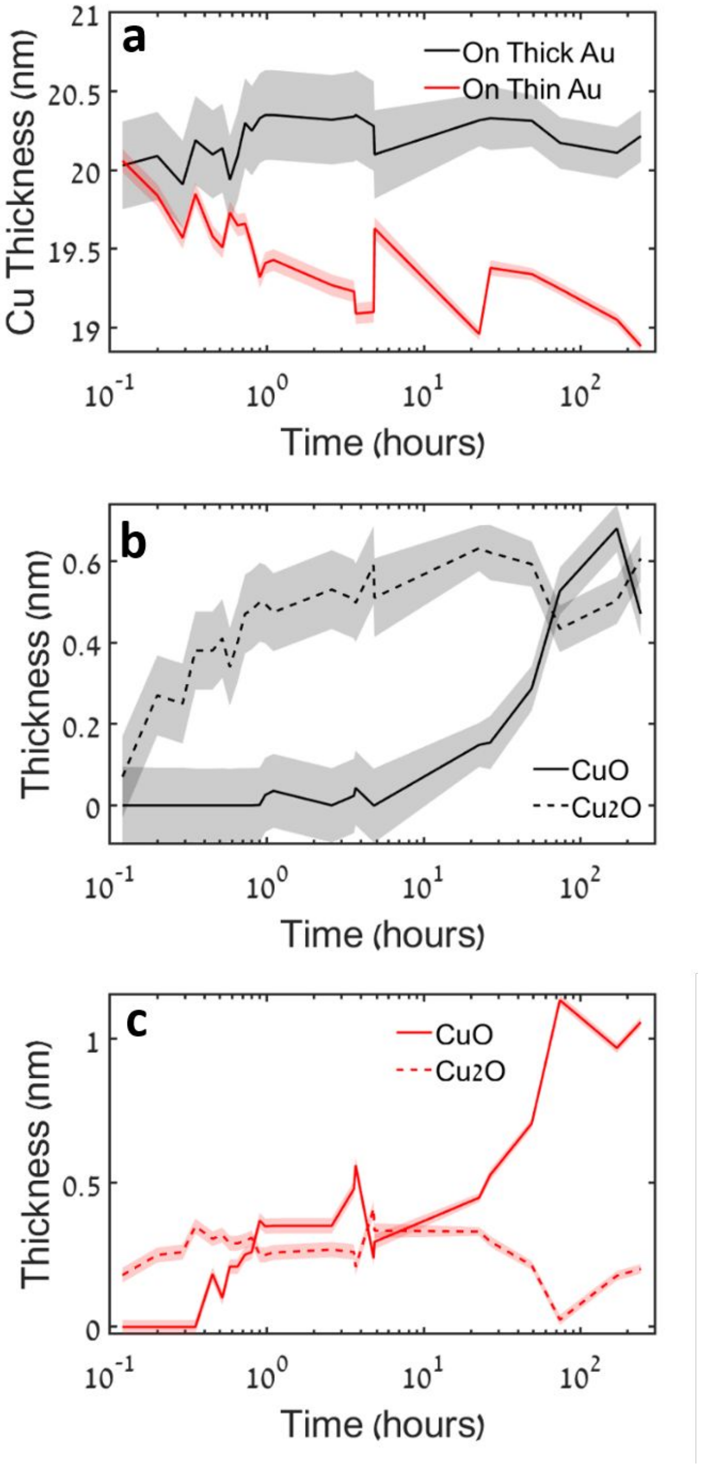

Figure S8 – Ellipsometry. *Ellipsometry measurement analysis plotting thickness as a function of time for metallic Cu, on both thick and thin Au regions (a) and for CuO and Cu_2O growing on the Cu film deposited over either thick (**b**) or thin (**c**) Au capping layers.*

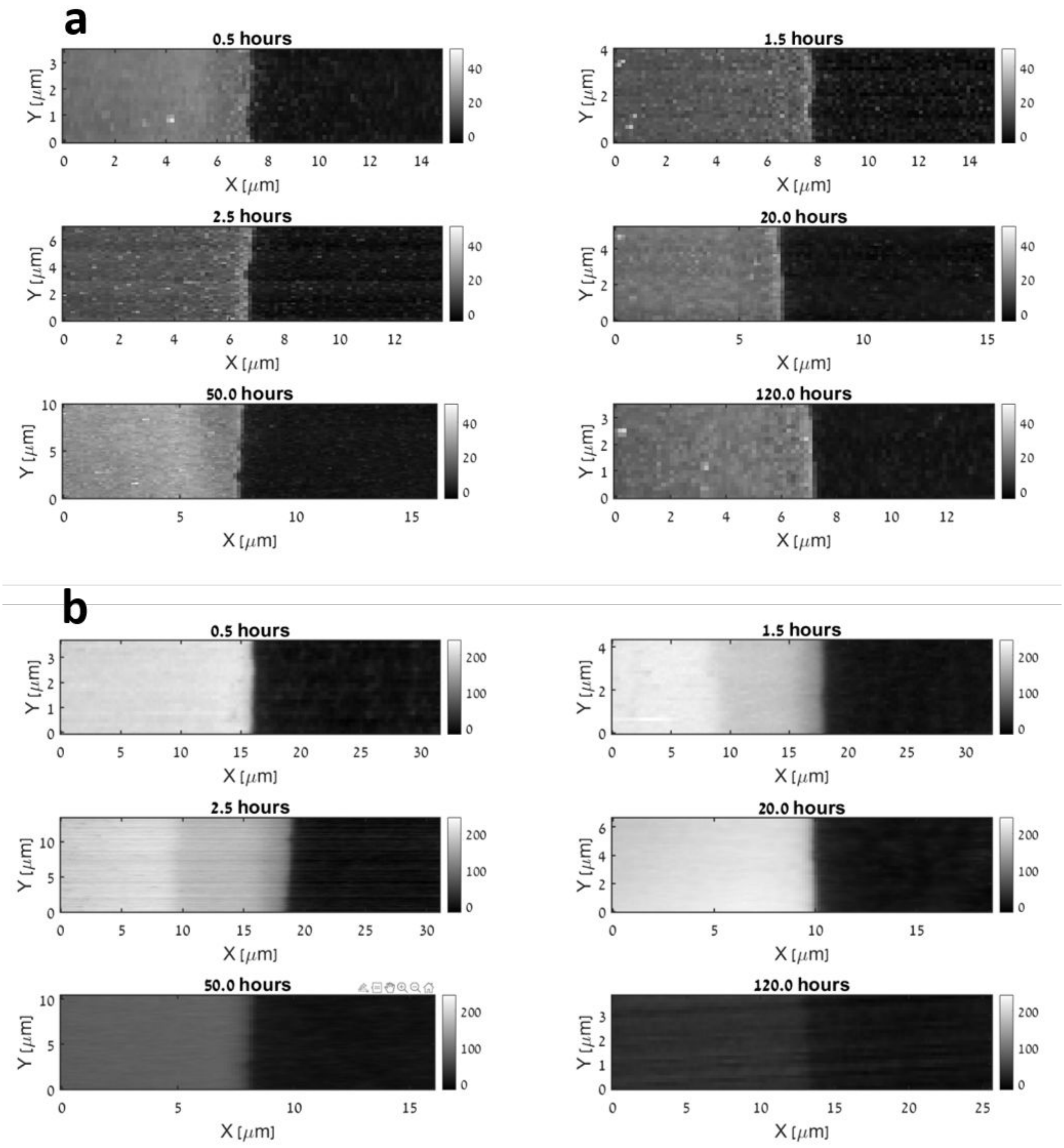

Figure S9 – Atomic Force Microscopy measurements. *Representative 2D topography (**a**) and CPD (**b**) scans for increasing times following exposure and oxidation at strip 2 of the wedge sample. The grayscale color bar values for height (**a**) are given in nanometers, and for CPD (**b**) are given in millivolts.*

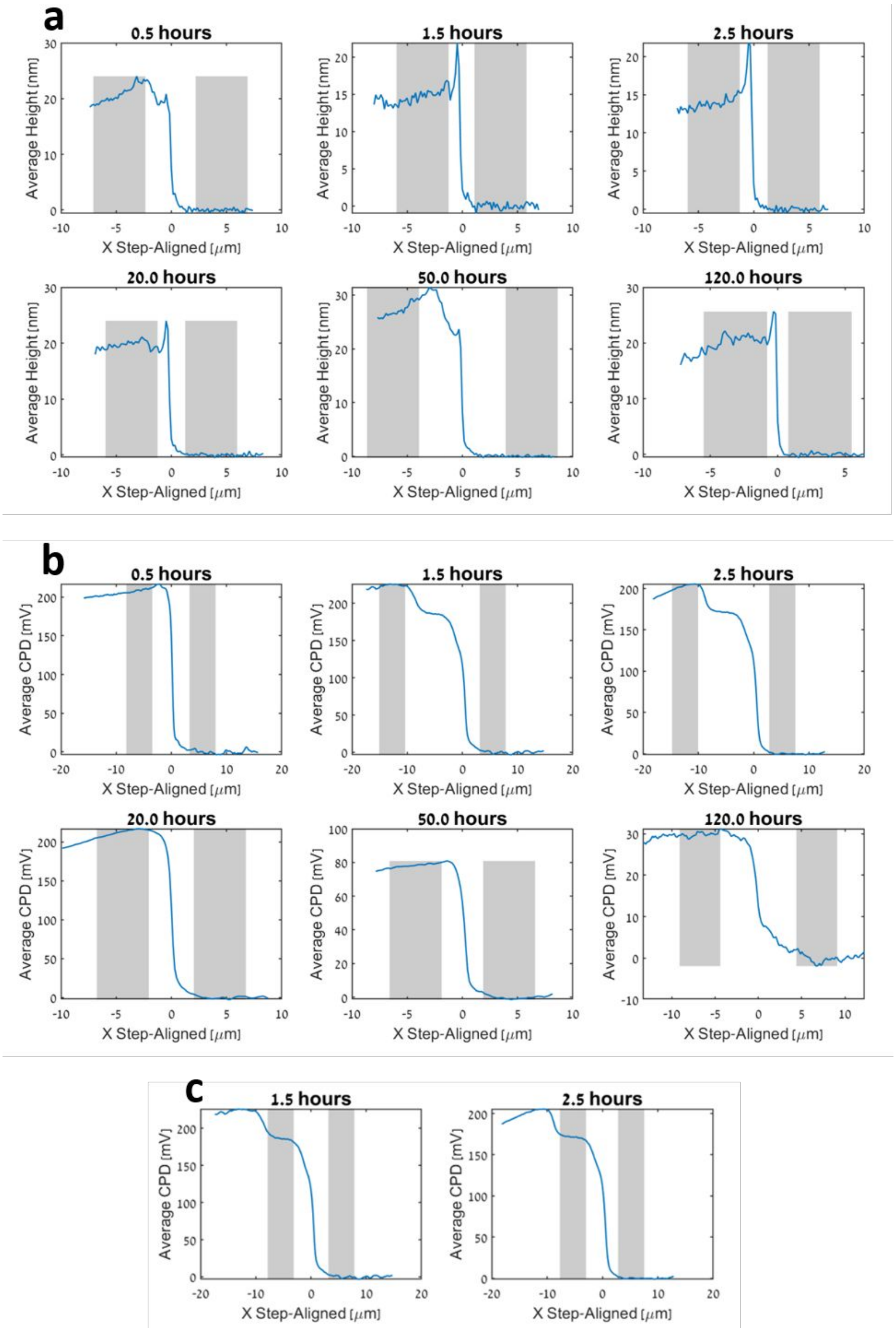

Figure S10 – Atomic Force Microscopy. *Representative averaging windows (shaded areas) used to calculate step height from average topography (**a**) and CPD (**b**) step profiles, obtained using KPFM, for increasing time points following exposure and oxidation at strip 2 of the wedge sample. Shoulder region CPD sub-step heights (c) were obtained similarly, when detected.*

$Cu/Cu_2O/CuO$ characterization by the XPS

X-ray photoelectron spectroscopy (XPS) measurements were performed using Kratos AXIS Supra spectrometer (Kratos Analytical Ltd., Manchester, U.K.) with Al Kα monochromatic radiation X-ray source (1486.6 eV). The XPS spectra were acquired with a takeoff angle of 90° (normal to analyzer); vacuum condition in the chamber was 2×10^{-9} Torr. The high-resolution XPS spectra were measured with pass energy of 20 and 0.1 eV step size. The binding energies were calibrated using C 1s peak energy as 285.0 eV. Data was collected and analyzed by using ESCApe processing program (Kratos Analytical Ltd.) and Casa XPS (Casa Software Ltd.).

Copper oxidation states which were measured by the XPS discussed in literature in many experiments. Generally, the Cu 2p doublet has a well-resolved chemical shift between Cu/Cu^{+} and Cu^{2+} peaks. Then the Cu^{2+} (CuO) peak shows the separate doublet on 935 and 955 eV the Cu^{+} (Cu_2O) has a similar binding energy as metallic Cu^{0} (932 and 952 eV). According to the Cu 2p core level measurements the Cu^{2+} (CuO) comprises about 10% of total Cu.

Usually, the additional measurement of X-ray induced Auger Cu LMM peak gives more specific signature for distinguishing the $Cu/Cu_2O/CuO$. In our case both Cu^{0} and Cu^{+} have appeared on the surface we use the Cu LMM and O 1s peaks to assume the ratio between these species.

For Cu LMM the peak on 569.6 eV supposed to be a Cu_2O, the calculated ratio Cu_2O/CuO is about 1.5. Analyzing the O 1s core peak we can see the three main components: 529.9 eV which should be assigned to CuO, 530.3 eV for Cu_2O and peaks on 531-532 eV which represent the O-C, -OH bonding. The ratio calculation for ratio Cu_2O/CuO is about 1.6 which is agree with Cu LMM line estimation.

Lucile Martin, Hervé Martinez, Delphine Poinot, Brigitte Pecquenard, Frédéric Le Cras. Comprehensive X-ray photoelectron spectroscopy study of the conversion reaction mechanism of CuO in lithiated thin film electrodes. *Journal of Physical Chemistry C, 2013, 117, 4421-4430.*

Z. Swiatkowska-Warkocka, M. S. Shakeri, O. Polit, J. Gurgul, M. Biesiadecka, A. Dziedzic, P. Pawlik, andJ.Kot. Surface Modification of CuO/Cu2O/Cu Composite Particles with Ag by Pulsed Laser Irradiationof Suspension and Their Antimicrobial Potential. *Journal of Physical Chemistry C, 2025, 129, 12953−12965*

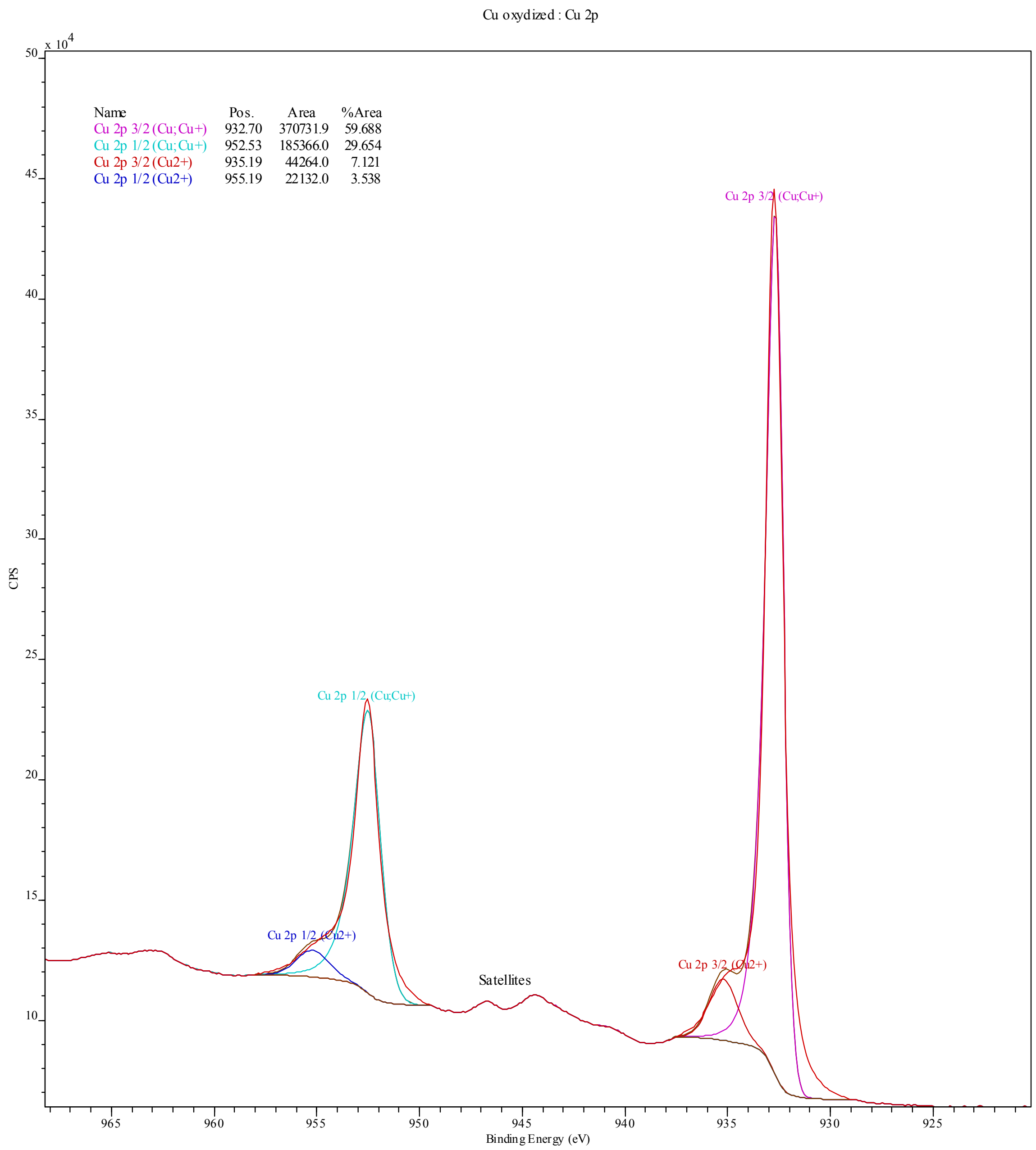
Cu oxydized : Cu 2p
x 10^4
Name Pos. Area %Area
Cu 2p 3/2 (Cu; Cu+) 932.70 370731.9 59.688
Cu 2p 1/2 (Cu; Cu+) 952.53 185366.0 29.654
Cu 2p 3/2 (Cu2+) 935.19 44264.0 7.121
Cu 2p 1/2 (Cu2+) 955.19 22132.0 3.538
Cu 2p 3/2 (Cu;Cu+)
Cu 2p 1/2 (Cu;Cu+)
Cu 2p 1/2 (Cu2+)
Satellites
Cu 2p 3/2 (Cu2+)
CPS
Binding Energy (eV)
50
45
40
35
30
25
20
15
10
965
960
955
950
945
940
935
930
925

Cu oxydized : Cu LMMa

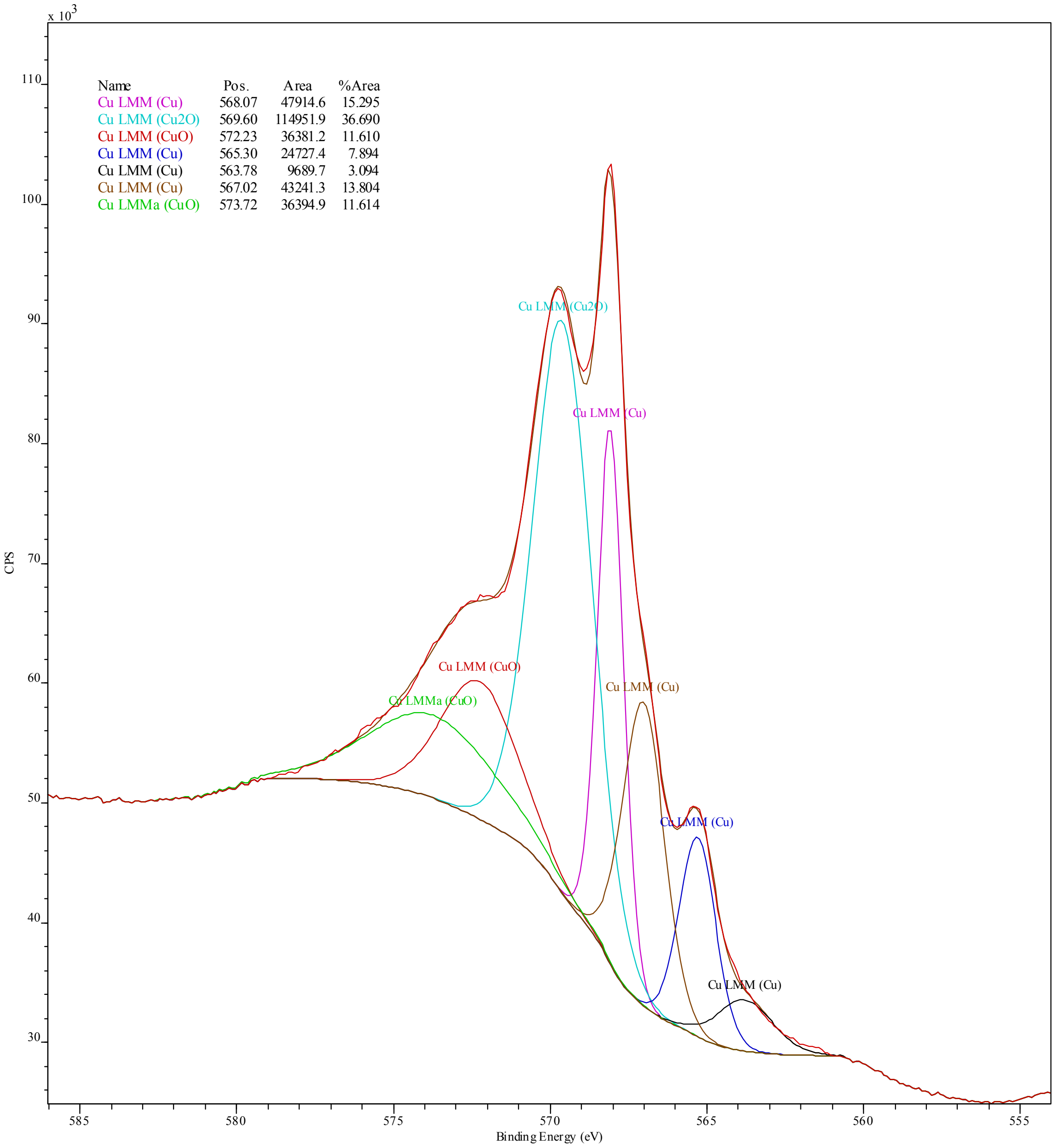

Cu oxydized : O 1s

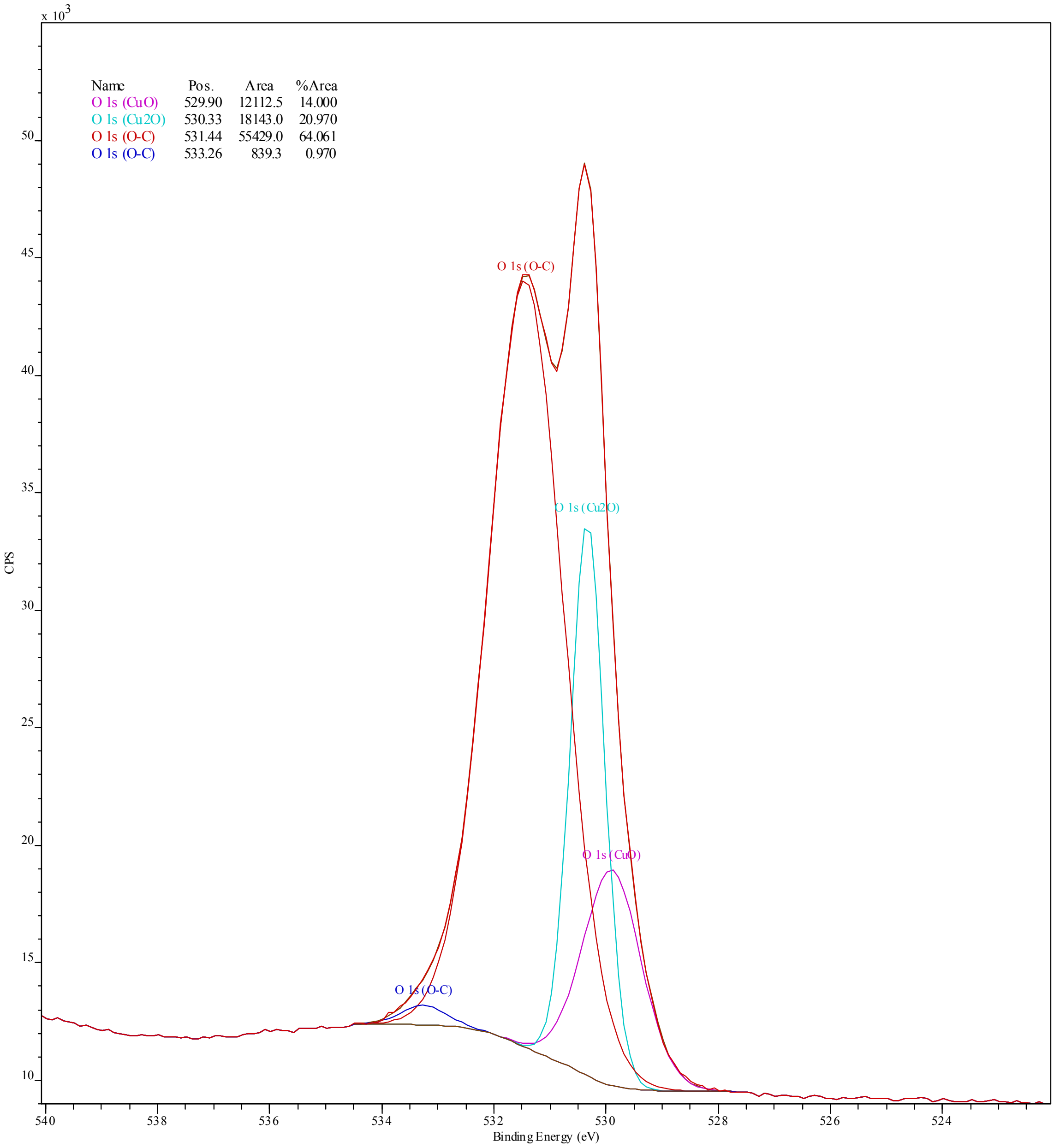

Supplement: Supplementary file 1 [file nn5c21063_si_001.pdf]
